# SCIP: software for efficient clinical interpretation of copy number variants detected by whole-genome sequencing

**DOI:** 10.1007/s00439-022-02494-1

**Published:** 2022-11-14

**Authors:** Qiliang Ding, Cherith Somerville, Roozbeh Manshaei, Brett Trost, Miriam S. Reuter, Kelsey Kalbfleisch, Kaitlin Stanley, John B. A. Okello, S. Mohsen Hosseini, Eriskay Liston, Meredith Curtis, Mehdi Zarrei, Edward J. Higginbotham, Ada J. S. Chan, Worrawat Engchuan, Bhooma Thiruvahindrapuram, Stephen W. Scherer, Raymond H. Kim, Rebekah K. Jobling

**Affiliations:** 1 Ted Rogers Centre for Heart Research, Cardiac Genome Clinic, The Hospital for Sick Children, Toronto, ON Canada; 2 Division of Clinical and Metabolic Genetics, The Hospital for Sick Children, Toronto, ON Canada; 3 The Centre for Applied Genomics, The Hospital for Sick Children, Toronto, ON Canada; 4 Program in Genetics and Genome Biology, The Hospital for Sick Children, Toronto, ON Canada; 5 CGEn, The Hospital for Sick Children, Toronto, ON Canada; 6 MIT Sloan School of Management, Massachusetts Institute of Technology, Cambridge, MA USA; 7 Department of Pathology, The University of Texas MD Anderson Cancer Center, Houston, TX USA; 8 Genome Diagnostics, Department of Paediatric Laboratory Medicine, The Hospital for Sick Children, Toronto, ON Canada; 9 Department of Molecular Genetics and the McLaughlin Centre, University of Toronto, Toronto, ON Canada; 10 Fred A. Litwin Family Centre in Genetic Medicine, University Health Network, Toronto, ON Canada; 11 Department of Medicine, University of Toronto, Toronto, ON Canada

## Abstract

Copy number variants (CNVs) represent major etiologic factors in rare genetic diseases. Current clinical CNV interpretation workflows require extensive back-and-forth with multiple tools and databases. This increases complexity and time burden, potentially resulting in missed genetic diagnoses. We present the Suite for CNV Interpretation and Prioritization (SCIP), a software package for the clinical interpretation of CNVs detected by whole-genome sequencing (WGS). The SCIP Visualization Module near-instantaneously displays all information necessary for CNV interpretation (variant quality, population frequency, inheritance pattern, and clinical relevance) on a single page—supported by modules providing variant filtration and prioritization. SCIP was comprehensively evaluated using WGS data from 1027 families with congenital cardiac disease and/or autism spectrum disorder, containing 187 pathogenic or likely pathogenic (P/LP) CNVs identified in previous curations. SCIP was efficient in filtration and prioritization: a median of just two CNVs per case were selected for review, yet it captured all P/LP findings (92.5% of which ranked 1st). SCIP was also able to identify one pathogenic CNV previously missed. SCIP was benchmarked against AnnotSV and a spreadsheet-based manual workflow and performed superiorly than both. In conclusion, SCIP is a novel software package for efficient clinical CNV interpretation, substantially faster and more accurate than previous tools (available at https://github.com/qd29/SCIP, a video tutorial series is available at https://bit.ly/SCIPVideos).

**Supplementary Information:**

The online version contains supplementary material available at 10.1007/s00439-022-02494-1.

## Introduction

Deletions or duplications of human genomic regions are collectively termed copy number variants (CNVs). CNVs range in size from 50 base-pairs (bp) to mega base-pairs (Mb). CNVs can exist as benign variations, e.g., > 20,000 CNVs with an allele frequency > 1% have been catalogued (Collins et al. 2020; Feuk et al. 2006; Iafrate et al. 2004; MacDonald et al. 2014; Sebat et al. 2004); however, they are also a major contributor to genetic disease as numerous contiguous gene syndromes have been documented (Amberger et al. 2019; Yuen et al 2017; Cerruti Mainardi 2006; Costa et al. 2022; McDonald-McGinn et al. 2015; Oskoui et al. 2015; Pereira and Marion 2018; Zarrei et al. 2019). Whole-gene deletions and intragenic deletions or duplications can cause loss-of-function, while whole-gene duplications result in local triploidy. To date, > 300 genes have been curated by the ClinGen Consortium (Rehm et al. 2015) as haploinsufficient.

Whole-genome sequencing (WGS) is increasingly being recommended as a first-line test for suspected rare genetic disorders (Lionel et al. 2018; Manickam et al. 2021; Marshall et al. 2020; NICUSeq Study Group et al. 2021). One major advantage of WGS is the ability to detect single-nucleotide variants (SNVs), indels, CNVs, and copy-neutral structural variants (SVs) genome-wide in a single test. Furthermore, unlike karyotyping and chromosomal microarray analysis (CMA), which have lower bounds on the CNV sizes they can detect, WGS identifies CNVs of all sizes. In recent studies, WGS was found to have superior sensitivity in detecting CNVs compared with CMA (Gross et al. 2019; Jiang et al. 2013; Trost et al. 2018). CNV detection in paired-end WGS data is supported by three major types of evidence: read depth (Abyzov et al. 2011; Handsaker et al. 2011; Zhu et al. 2012), paired-end reads with abnormal insert size and/or orientation, and split reads (Figure S1), with the latter two commonly referred to as anomalous reads (Chen et al. 2016).

Several factors make the clinical interpretation of CNVs challenging. Caused by the relatively higher false detection rate of CNVs compared with SNVs and indels, greater emphasis must be placed on variant quality assessment. Tools such as the Integrative Genomics Viewer (IGV) (Thorvaldsdóttir et al. 2013) are used to visually inspect read depth and/or anomalous reads at the putative CNV. However, this can be time-consuming, as it can take one minute or more per CNV for IGV to display alignments (depends on read depth and size). In addition, visualization relative to other annotations is essential to interpret CNVs. During this step, an analyst must query multiple databases, e.g., gnomAD-SV (Collins et al. 2020) and DGV (MacDonald et al. 2014) for benign variation, ClinGen dosage sensitivity curations for haploinsufficient (HI) and triplosensitive (TS) regions, genome browsers for genes, and ClinVar (Landrum et al. 2018) and DECIPHER (Firth et al. 2009) for pathogenic variation. This process is complex and error-prone, as it involves back-and-forth maneuvers and synthesizing evidence across multiple webpages.

Several publicly available tools have been developed to address these inefficiencies. ClinSV is a CNV/SV analysis pipeline that uses custom IGV tracks to display binned read depth and mapping quality, anomalous reads, and variants from both an internal database and DGV (Minoche et al. 2021). Another tool, samplot, pre-generates read depth and anomalous read plots for manual review of CNV quality (Belyeu et al. 2021). CNspector is an interface for interactive viewing of copy number changes at scales from single-exon to genome-wide, particularly suitable for cancer genomes (Markham et al. 2019). CNVxplorer allows users to query biological annotations (e.g., pathway enrichment, KO models, regulatory regions) of a CNV, but could not be used for variant quality assessment (Requena et al. 2021). AnnotSV (with visualization provided by knotAnnotSV) is a web-based tool that performs annotation, prioritization, and visualization for human CNVs and SVs (Geoffroy et al. 2021). However, it is not capable of visualizing variant quality or incorporating it for prioritization. In summary, some of these tools are suitable for CNV quality assessment, while others are designed to explore clinical relevance; however, no publicly available tool exists that allows for investigation of both aspects simultaneously in a unified and integrated environment.

Here we present the Suite for CNV Interpretation and Prioritization (SCIP), which provides a Visualization Module that near-instantaneously displays all information necessary for clinical CNV interpretation. The Visualization Module is supported by a backend, providing variant filtration and prioritization. SCIP was rigorously evaluated using 1027 families ascertained for congenital cardiovascular disease and/or autism spectrum disorder (ASD). SCIP performed substantially better than a spreadsheet-based manual workflow and AnnotSV (Geoffroy et al. 2021).

## Materials and methods

### Computational requirements and implementation of SCIP

The SCIP backend was implemented at the high-performance computing facility of SickKids Research Institute. Each instance of the SCIP backend was run on a single core on a server with a 2.3-GHz Skylake Intel Xeon processer and 8 GB of memory (unless for CNVs > 10 Mb in size [up to 64 GB of memory was used]) running CentOS 7. Required software include Perl (v5.16), R (v3.5.1), samtools (v1.10), bedtools (v2.26), and tabix (v0.2.5). The Visualization Module was implemented on the analysts’ personal computers, running macOS Big Sur or Windows 10 using Google Chrome. Required software includes R, RStudio, and three R packages (shiny, DT, and plotrix) and their dependencies. See Supplementary Materials for details on generating the annotation files used by SCIP.

### The spreadsheet-based manual workflow for clinical CNV interpretation

After variant calling, all CNVs in a given sample were processed by an in-house annotation pipeline, which outputs a spreadsheet where each row is a CNV and each column is an annotation. The following types of annotation were included: variant quality, overlap with common (variant frequency > 1%) and rare CNVs in gnomAD-SV, DGV, and internal control databases, overlap with genes and curated dosage sensitive regions, gene constraint information (e.g., gnomAD and ExAC pLI Karczewski et al. 2020; Lek et al. 2016)), and gene–phenotype/disease association. This spreadsheet-based workflow was applied to both the CGC and MSSNG samples. For the MSSNG samples, an additional column indicates whether the CNV was considered as “high quality rare”.

The spreadsheet was reviewed by an analyst with an advanced degree in genetics, tasked with identifying potentially reportable CNVs. Filters, typically a combination of variant quality and gene constraints, were used for CNV prioritization. Regions that harbour candidates (putative CNVs that appears to be reportable based on the information in the spreadsheet, if their variant qualities are satisfactory, i.e., if they are true positive CNV calls) were visualized in IGV to assess read depth and anomalous reads. This process is time-consuming, as loading read alignments may take a long time (especially for large CNVs) and additional efforts may be required to inspect anomalous reads (particularly split reads). The analyst would consult various online tools to confirm the information in the spreadsheet and/or gather additional information. Classification was based on the synthesis of all available information, considering the patient’s clinical manifestation(s).

For the CGC samples, the CNVs identified by the analysts were further reviewed by a panel of clinical and molecular geneticists and genetic counsellors. For the MSSNG samples, the CNVs identified by the analysts were further reviewed by at least three clinical genetics experts with advanced degrees and/or postgraduate experience in human genetics.

### Cohorts used for the evaluation of SCIP

The CGC samples (*n* = 316 families) were sequenced at the Cardiac Genome Clinic of The Hospital for Sick Children for congenital cardiovascular diseases, primarily congenital heart defects. Participant recruitment and genome analysis procedures were described previously (Reuter et al. 2020). Briefly, the samples were sequenced on the Illumina HiSeq X or NovaSeq 6000 platforms at The Centre for Applied Genomics (TCAG) in Toronto, Canada to generate 150-bp paired-end reads at ≥ 30 × coverage. Reads were mapped to the GRCh37/hg19 reference genome using BWA (Li and Durbin 2009). CNVs were identified using ERDS (Zhu et al. 2012) and CNVnator (Abyzov et al. 2011) calls, with a window size of 500 bp. High-quality CNVs were identified as those detected by both methods with > 50% overlap (Trost et al. 2018). Manta was also used to identify CNVs based on anomalous reads (Chen et al. 2016).

We also analyzed WGS data from the MSSNG Project, which contains nearly 3000 families sequenced for autism spectrum disorder (Trost et al. 2022). These samples were analyzed by The Centre for Applied Genomics at The Hospital for Sick Children, aligned to GRCh38/hg38. CNVs were detected using the algorithms ERDS (Zhu et al. 2012) and CNVnator (Abyzov et al. 2011) based on a previously described workflow (Trost et al. 2018). A CNV was deemed rare if its frequency was < 1% in MSSNG parents and in 1000 Genomes Project population controls according to both algorithms. A CNV was deemed to be “high quality rare" if it was rare, was detected by both ERDS and CNVnator with at least 50% reciprocal overlap, and less than 70% of the CNV overlapped assembly gaps, centromeres, and segmental duplications. CNVs underwent at least three rounds of manual curation by experienced scientists to identify P/LP CNVs and reportable VUS that may be responsible for autism spectrum disorder. As families with no reportable CNV add little value in evaluating SCIP, we randomly excluded about 2/3 of such families from this study, resulting in a collection of 711 families.

CNVs in both cohorts were extensively curated. In both the CGC and MSSNG cohorts, P/LP CNVs were identified using the spreadsheet-based manual approach described above. The CGC and MSSNG cohorts collectively form the cohort used to evaluate SCIP. For this study, all previously identified P/LP CNVs were re-interpreted by an analyst using the ACMG/ClinGen guidelines (Yuen et al 2017; Riggs et al. 2020), ensuring that they met the threshold of LP (0.90 points). CNVs not meeting the threshold were downgraded to VUS.

This highly diverse and expertly curated cohort included 1,027 families, 316 aligned to hg19 and 711 aligned to hg38 (Figure S2a), among which 174 had one or more P/LP CNVs (Figure S2b). The P/LP CNVs were diverse: 121 deletions and 66 duplications (Table S1), size ranging from 2.51 kb to 77.01 Mb, a mixture of de novo and inherited variants, covered several recurrent regions (e.g., distal 1q21.1, 16p11.2, 22q11.2), and reached P/LP by different ACMG/ClinGen rule combinations (Figures S2c, S2d, and S2e).

### Comparison with AnnotSV

We also compared the performance of SCIP with a recently published CNV/SV prioritization tool, AnnotSV (Geoffroy et al. 2021). We selected 15 ASD cases with a good diversity of P/LP CNV type and size, including two cases with no P/LP CNV. For each case, we uploaded the full list of CNVs (the same list provided to the SCIP backend) to the AnnotSV web server (https://lbgi.fr/AnnotSV/runjob). The svtBEDcol option was set to 4. For phenotype-driven analysis, we used the HPO term HP:0000717 (autism). All other options were kept at default. We did not upload the optional SNV VCF file, as we found that it had little, if any, effect on prioritization. CNVs with a score of 4 or 5 were considered prioritized. Performance was measured by the number of prioritized CNVs requiring manual review, as well as the rank of the P/LP CNV (if any) among the reviewable CNVs.

## Results

### Implementation and software architecture of SCIP

**Fig. 1:**
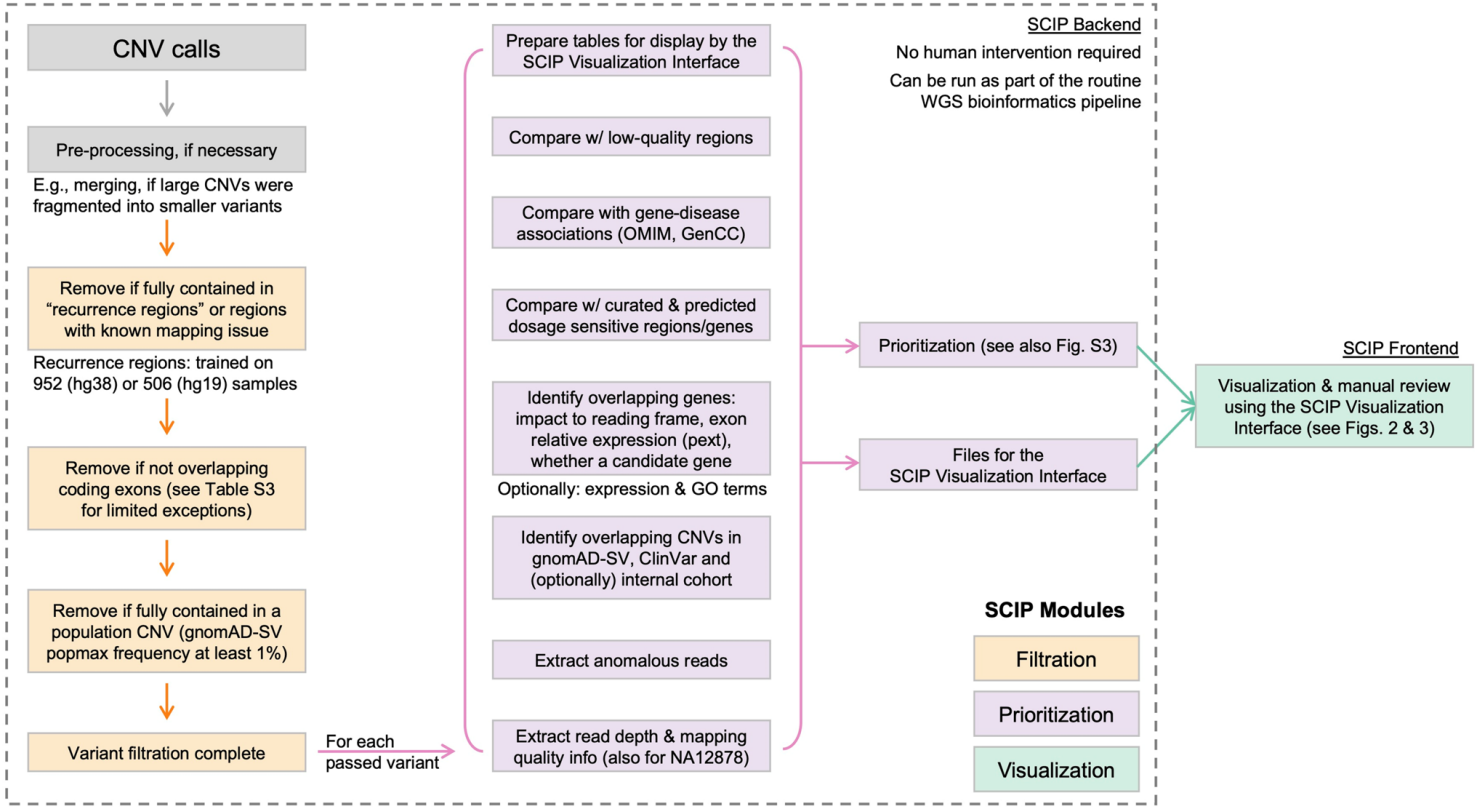
Overall Software Architecture of SCIP. SCIP is composed of three modules. The Variant Filtration and Prioritization Modules collectively form the SCIP backend (within the dotted rectangle). CNV calls, after necessary pre-processing (e.g., merging), pass through the three-step Variant Filtration Module (orange). The remaining variants are then analyzed by the Prioritization Module, which calculates a priority score (Figure S3) and generates files for the Visualization Module. User may also opt to perform their own filtering and skip the SCIP Variant Filtration Module

SCIP is composed of three modules: Variant Filtration, Prioritization, and Visualization. The Variant Filtration and Prioritization Modules together form the SCIP backend, while the Visualization Module is the frontend (Fig. 1). The backend was implemented using Perl and R, while the frontend was implemented as a R Shiny application. The SCIP backend can be implemented on a high-performance computing server or a personal computer, while the Visualization Module is implemented on the analyst’s personal computer. Files generated by the Prioritization Module must be available to the Visualization Module.

For a single sample, the minimum input file requirements are a list of CNVs and a BAM/CRAM alignment file. Optionally, if genome sequencing has been performed on multiple related individuals, CNVs detected in those individuals may be used as input for the purpose of inheritance analysis. SCIP requires annotations from external databases and the alignment file of a reference sample (e.g., NA12878, a widely used control sample (Zook et al. 2016)) (Table S3). Variants passing Filtration are processed by the Prioritization Module, then visualized in the Visualization Module, sorted by priority and size. SCIP is compatible with both hg19 and hg38 genome builds. SCIP is available on GitHub (https://github.com/qd29/SCIP). The SCIP GitHub webpage also includes step-by-step instructions (also see Supplementary Texts 1 and 2) and a demo of the SCIP Visualization Module. To further improve usability, a video tutorial series covering the setup and usage of SCIP is on YouTube at https://bit.ly/SCIPVideos.

### SCIP variant filtration and prioritization modules

CNVs are first processed by the SCIP Variant Filtration and Prioritization Modules (collectively, the SCIP backend; Fig. 1). While CNVs from all callers (based on read depth or anomalous reads) are acceptable, interval merging is necessary if large CNVs were broken into fragments (e.g., by gaps), as SCIP may not be able to handle substantial under-calling (see Discussion and Supplementary Materials).

Variant filtration has three steps for optimal efficiency. In the first step, CNVs fully contained in regions with known issues (gaps, centromeres, repeats) and recurrence regions (for details, see Supplementary Materials) are removed. In the second step, CNVs that overlap coding sequences are retained, in addition to CNVs that overlap genes with clinically relevant non-coding variation (Table S2). Finally, CNVs are removed if they are fully contained in population variations (i.e., same type of CNV seen in > 1% frequency in a gnomAD-SV population). The remaining CNVs are passed to the Prioritization Module. If users opt to perform their own filtration, they may start directly with the Prioritization Module.

The Prioritization Module (Table S3) annotates and calculates a priority score for each CNV. For a given CNV, the following types of annotations are generated by the Prioritization Module: (1) overlapping CNVs in gnomAD-SV (including their allele frequencies), (2) overlapping CNVs in ClinVar (including their pathogenicity interpretations, associated conditions, allele origins, and gene contents), (3) overlapping CNVs in the internal cohort (if provided), (4) overlapping ClinGen dosage sensitive regions and genes, and (5) overlapping genes (including whether the overlap is full or partial, strand information, associated conditions provided by OMIM and GenCC, gnomAD gene constraints, exons and transcripts affected by the CNV, and exon-level relative expression data [i.e., pext scores]).

It is important to note that some annotation files used by the Prioritization Module, specifically (1) ClinGen dosage sensitivity curations, (2) ClinVar CNV information, (3) OMIM and GenCC gene-disease associations, and (4) the non-coding pathogenic regions, require periodic updates. The current guidelines recommend updating items 1–3 at least quarterly (Austin-Tse et al. 2022), and update item 4 if new pathogenic non-coding regions are discovered. See Table S3 for additional instructions.

The priority score is the sum of two components: clinical relevance and adverse information. Lower scores denote higher priority. The clinical relevance score is based on whether the CNV overlaps any ClinGen-curated dosage sensitive region, genes with substantial loss-of-function constraints, and/or genes associated with genetic conditions. While the theoretical range of the clinical relevance score is from 1 to 99, currently we only use 1–5 and 99 for deletions, and 1–7 and 99 for duplications. The default clinical relevance score (for CNVs without known clinical relevance) is 99. In other words, a CNV with any clinical relevance will have a score between 1 and 5 for deletions, and 1 and 7 for duplications. For example, a deletion that fully contains a ClinGen-curated HI region has a clinical relevance score of 1. The adverse information score is based on whether there is evidence against the CNV being true and/or pathogenic. This score is binary: if adverse information exists, the score will be 100; otherwise, the score will be zero. A priority score below 99 (with clinical relevance and without adverse information, currently 1–5 for deletions and 1–7 for duplications) denotes high priority. A priority score of 99 (without clinical relevance or adverse information) denotes moderate priority. A priority score above 99 (any clinical relevance and with adverse information) denotes low priority. Only CNVs with high priority need further review. The scoring scheme was described in detail in Figure S3. The Prioritization Module also generates files to be used by the Visualization Module for rendering tables and plots. The SCIP backend is fully programmatic and can be seamlessly integrated into the clinical WGS bioinformatics pipeline.

### Overview of the SCIP visualization module/interface

The SCIP Visualization Module is a web-based graphical user interface (GUI) developed using the R Shiny package. Parameters are specified in a text configuration file (Table S5).

**Fig. 2:**
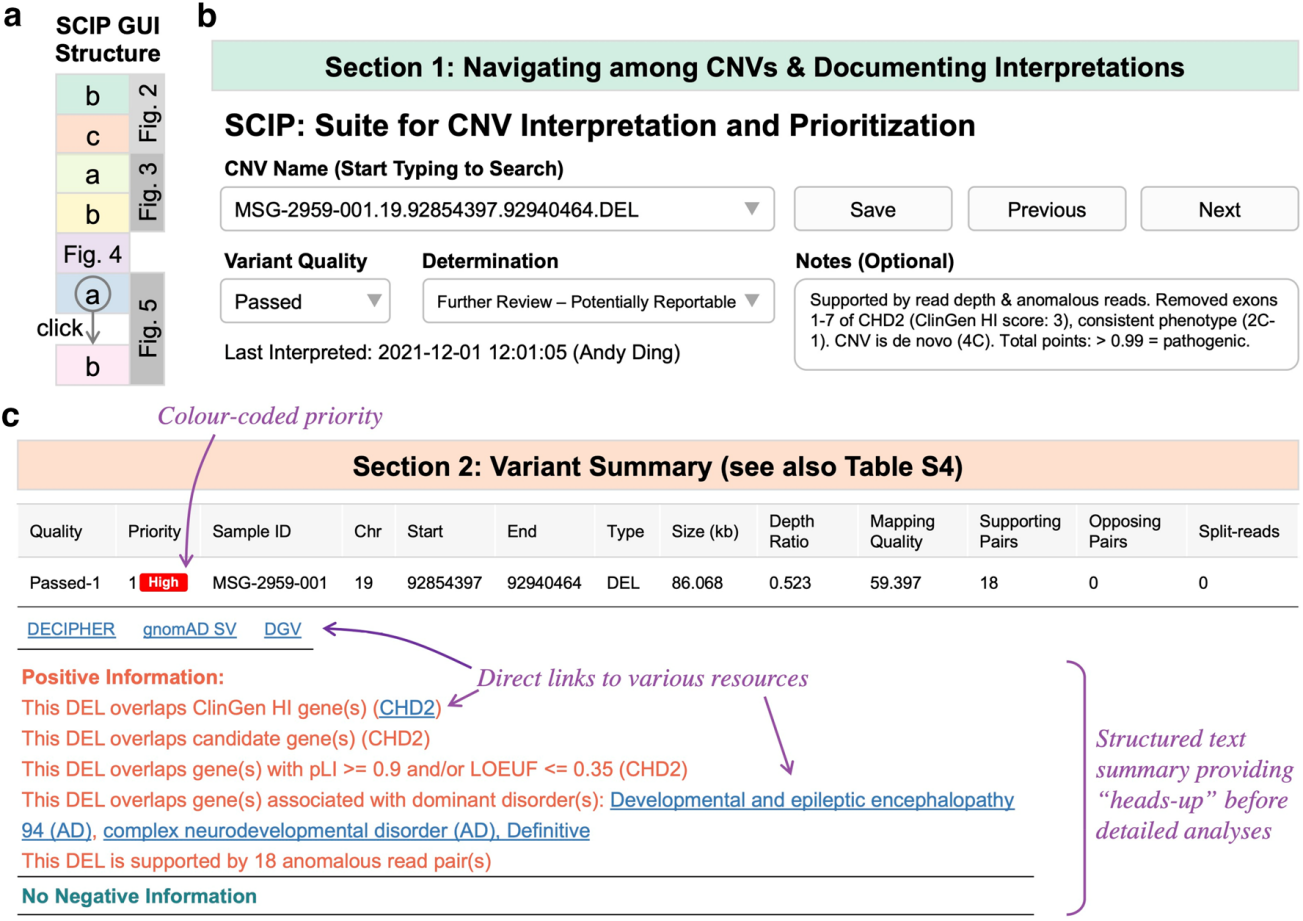
SCIP Visualization Module, Part 1. **a** Schematic of the SCIP Visualization Module. This panel provides an overview of the SCIP Graphical User Interface (GUI, i.e., the Visualization Module). This panel illustrates that the SCIP Visualization Module displays multiple sections sequentially. The details of these sections are shown in additional figure panels (the names and colour codes of which are indicated). One of the sections (Sect. 6.1) of the SCIP Visualization Module is toggled by a mouse click (for more details, see Fig. 5), as indicated by the “click” wording in this panel. **b** Sect. 1 allows navigation through CNVs, either using the searchable CNV Name drop-down menu or the previous/next buttons. A user may view, modify, or enter interpretations. **c** The Variant Summary section offers an overview of a CNV, facilitating precision analysis. For this deletion, this section displays that it overlaps *CHD2*, a curated HI gene, and is well-supported by anomalous reads

The GUI contains six sections (Fig. 2a), logically organized based on the manual workflow and the ACMG/ClinGen CNV interpretation guidelines (Riggs et al. 2020). The first section (Fig. 2b) allows the user to navigate through the CNVs in a sample, view previous interpretations (if any), and enter or modify interpretations. Interpretations are centrally stored in a text file.

The second section (Fig. 2c) provides an overview of a given CNV, intended to improve the efficiency of interpretation by highlighting key evidence. Priority calculated by the Prioritization Module is colour-coded (Figure S3). Structured sentences list positive (e.g., fully contains HI region) and negative (e.g., substantial overlap with population variants) findings (Table S4). In addition, the ratio of median read depth within vs. flanking the variant, median mapping quality within the variant, and a quality score (guidance only, Supplementary Materials) are displayed in the table.

**Fig. 3:**
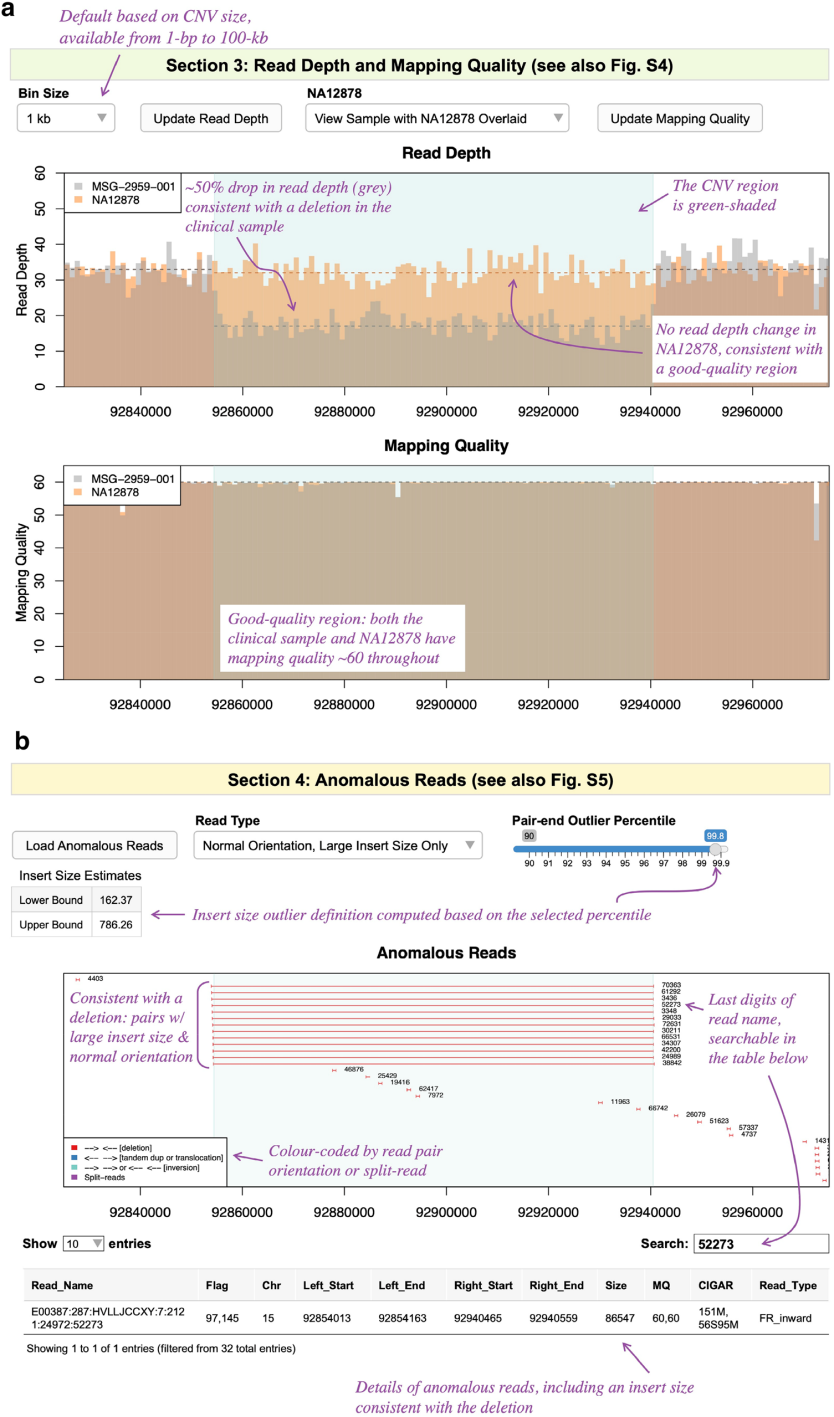
SCIP Visualization Module, Part 2. **a** Sect. 3 plots binned read depth and mapping quality information. **b** Sect. 4 plots anomalous reads in the region flanking the variant. The upper/lower bound of insert size (for outlier detection) is set as 99.5/0.5-percentile of that of all read pairs by default and is adjustable. A user may view specific kinds of anomalous reads depending on the CNV type. For example, this plot only shows read pairs with normal orientation but unusually large insert size (expected for a deletion). Part of their names is displayed alongside the reads, which are searchable in the table below. Together with Sect. 3 (panel a), they offer a more efficient alternative to IGV in assessing CNV quality. Purple text in the figure highlights additional features of the interface

The third and fourth sections facilitate variant quality assessment, as a more efficient alternative to IGV. The third section (Figs. 3a and S4) plots binned read depth and mapping quality of the CNV and its flanking regions (50% of variant size, minimum 100 kb, on both sides). All plots in the Visualization Module are interactive, allowing parameter adjustments and zooming in and out. By default, the plots have data from NA12878 overlaid (as a reference) but may be adjusted to display the clinical sample only. Common spurious read depth changes (e.g., regions with mapping issues) visible in the reference sample can be identified and excluded.

The fourth section (Figs. 3b and S5) displays colour-coded anomalous reads: read pairs with inward (normal) orientation but very large or small insert size (red), read pairs facing outward (blue) or in the same direction (cyan), and split reads (purple). Due to technical differences, the number of supporting reads plotted may be slightly lower than displayed in the second section. When zoomed-in, partial read names are displayed next to the reads, which are searchable in the accompanying table. This table shows details of anomalous reads, which is helpful when determining the exact CNV breakpoints. Using the Read Type drop-down menu, users may view a subset of anomalous reads specific to the variant type (Figures S1 and S5), e.g., inward pairs with large insert size when interpreting a deletion.

**Fig. 4:**
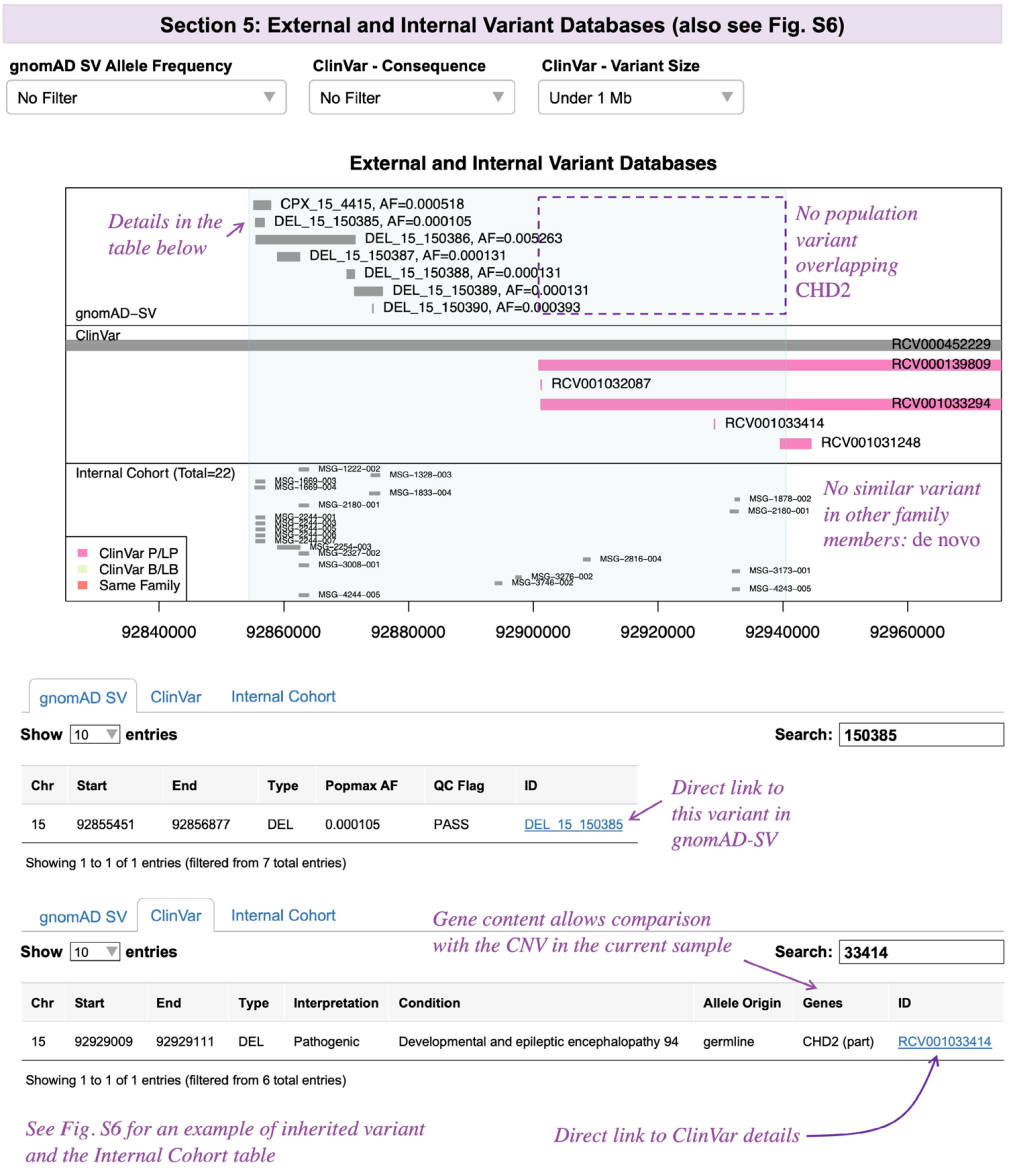
SCIP Visualization Module, Part 3. The External and Internal Variant Databases section compares the variant-of-interest with known CNVs, as well as the internal cohort (if provided). In the gnomAD-SV panel, names and popmax allele frequencies are displayed next to the variants. This is supplemented by a table with links to gnomAD-SV. For this CNV, no gnomAD-SV variants overlapping *CHD2* (purple box) were observed. Filtering of variants by popmax frequency is available. ClinVar variants are colour-coded by consequence (see legend) and may be filtered by consequence or size. The accompanying table displays gene content of the ClinVar variants (including whether full or partial overlap), allowing comparison with the CNV-of-interest. In the Internal Cohort panel, variants from the same family as the proband are coloured in red. There are no red-coloured similar-sized variants in this panel (despite trio sequenced), indicating that the variant is de novo

After a putative CNV is determined to be of satisfactory quality using Sects. 3 and 4, a user will then proceed to Sect. 5 (Figs. 4 and S6). This section visualizes the CNV-of-interest relative to known CNVs, allowing the identification of benign population variants (gnomAD-SV (Collins et al. 2020)) and recurrent pathogenic variants that have been interpreted previously (ClinVar (Landrum et al. 2018)). If the user provided CNV data from other family members, their CNVs will be coloured in red in the Internal Cohort panel, facilitating the study of inheritance patterns. Figure 4 shows a de novo variant (trio sequenced where the variant was absent in parents), and Figure S6 demonstrates a maternally inherited variant. When zoomed-in, IDs are displayed alongside the variants, which are searchable in the tables below that contain additional information (e.g., links to databases, gene content for ClinVar variants).

**Fig. 5:**
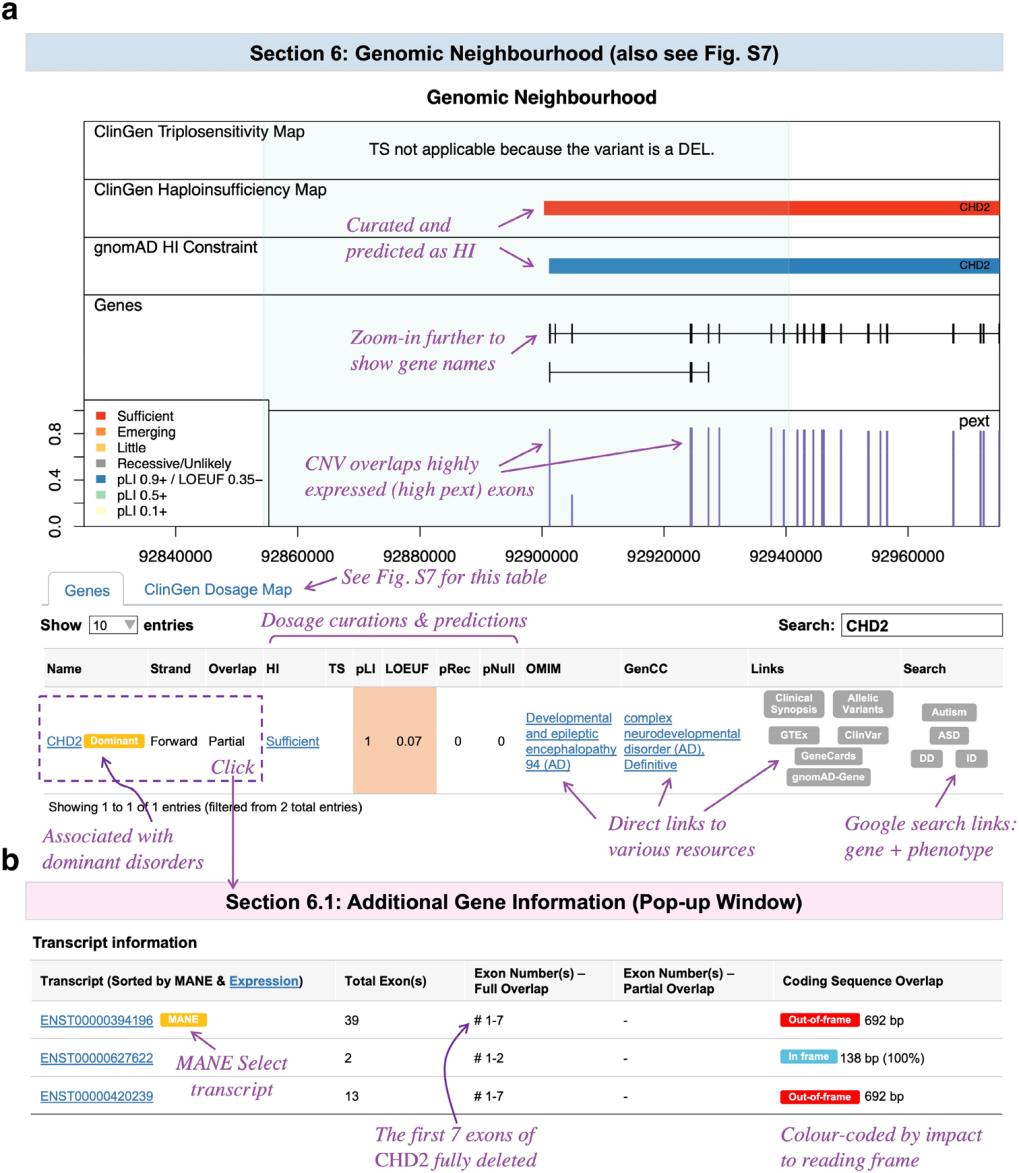
SCIP Visualization Module, Part 4. **a** The Genomic Neighbourhood section plots dosage sensitivity curations and constraints, genes, and pext (relative exon expression) scores. Dosage information is colour-coded (see legend). The Genes table comprises a wealth of information, including links to external resources (e.g., OMIM clinical synopsis and allelic variants pages, GTEx, GeneCards, Google search terms). **b** The Transcript Information table shows exons in biologically relevant transcripts affected by the CNV. This pop-up table can be toggled by clicking one of the first three columns of the Genes table. The queried CNV removed exons 1–7 of the MANE Select transcript of *CHD2*, supporting its pathogenicity

The sixth section (Figs. 5 and S7) provides the biological and clinical context of the CNV relative to genomic annotations. Tailored to the ACMG/ClinGen guidelines (Riggs et al. 2020), the following are plotted: dosage sensitivity curations (ClinGen) and constraints (gnomAD pLI (Karczewski et al. 2020)), coding exons (see Table S2 for limited exceptions), and relative exon expression (gnomAD pext (Cummings et al. 2020)). The pext score is helpful to evaluate the biological relevance of affected exons (Abou Tayoun et al. 2018). The accompanying tables contain a wealth of information, including direct links to databases and Google search terms (gene + phenotype), to facilitate additional exploration of genes overlapping the CNV. Further, the first three columns of the Genes table function as clickable buttons that lead to a pop-up window containing affected exon information (Fig. 5b). This is helpful in investigating the impact of intragenic CNVs on reading frames.

Taken together, the SCIP Visualization Module offers a logically organized workflow, guiding users through a thorough clinical interpretation of CNVs—quality assessment, relationship to known benign and pathogenic variants, inheritance, and genomic context. Taking advantage of being web-based, the interface also provides many direct links, e.g., to DGV, OMIM, GeneCards, and Google. These links allow users to explore databases rapidly and seamlessly, saving time and reducing errors. A video walkthrough of the SCIP Visualization Module can be found on YouTube at https://bit.ly/SCIPVideos (video #3).

To further demonstrate how SCIP facilitates efficient identification of clinically reportable CNVs and exclusion of non-relevant putative CNVs, we presented four typical use cases of SCIP in Supplementary Text 3 (a pathogenic deletion, a pathogenic duplication, a population variant, and a CNV not affecting biologically relevant transcript).

### Computational performance of the SCIP backend

We evaluated the computational burden of the SCIP backend using a single core on a server with a 2.3-GHz Skylake Intel Xeon processer. Variant filtration took 0.18 min per sample (i.e., 0.58 min per 10,000 CNVs), while the Prioritization Module used a median of 10.47 min (IQR: 10.06, range 0.52–136.77) per sample. Files generated for the Visualization Module occupied a median of 64.90 MB (IQR: 64.67, range 4.04–1039.49) of storage per sample. While RAM usage was not monitored, 8 GB was adequate for the Variant Filtration Module and most CNVs processed by the Prioritization Module (although CNVs > 10 Mb may require more than 8 GB of RAM). These results indicate that the SCIP backend had a minimal computational burden. Furthermore, the Variant Filtration (by chromosome) and Prioritization Modules (by CNV) support parallelization, allowing speed improvements when resources permit.

### SCIP was efficient at variant filtration and prioritization

**Fig. 6:**
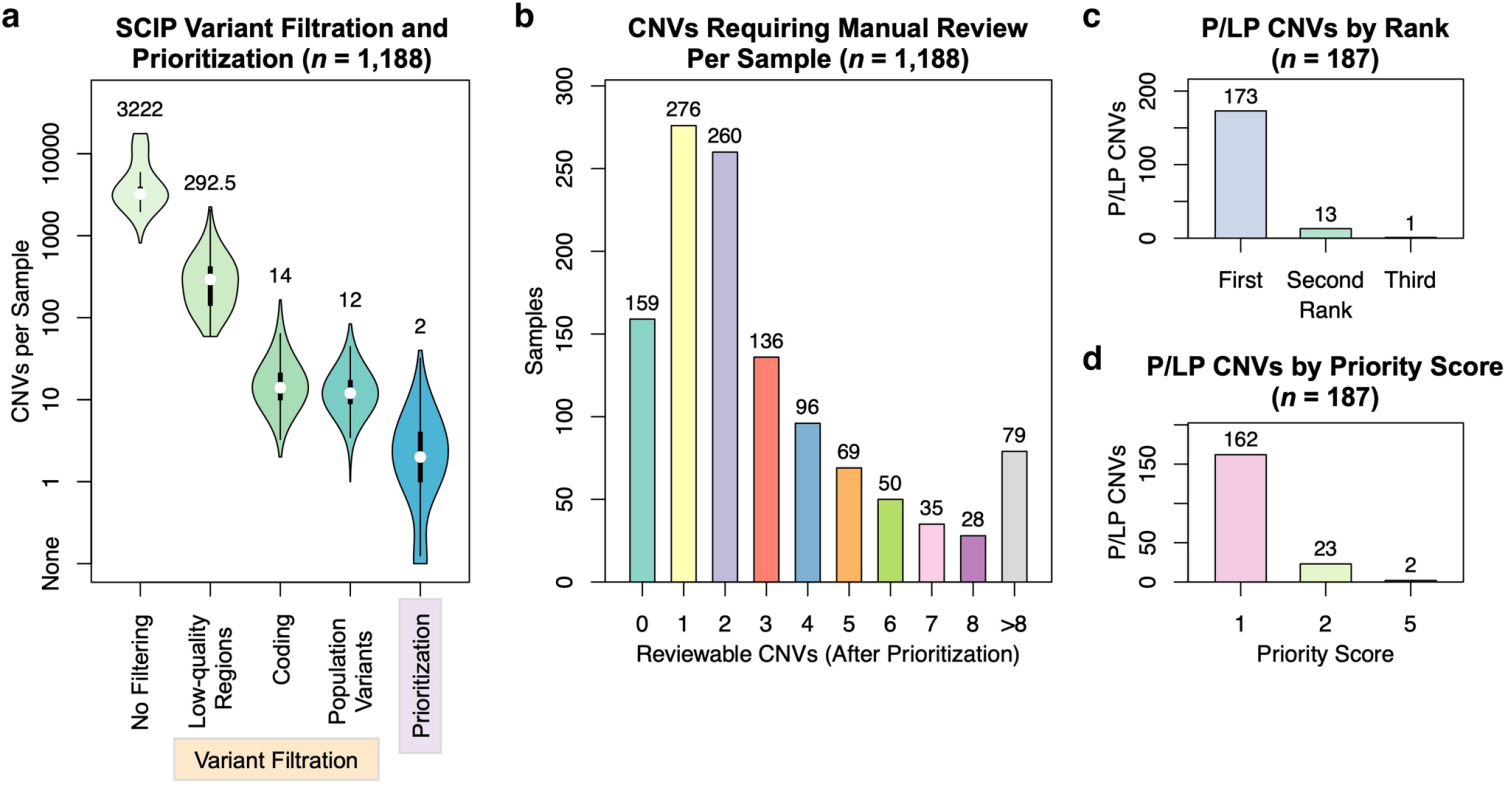
SCIP was Highly Efficient at Filtration and Prioritization of P/LP CNVs. **a** The SCIP Variant Filtration and Prioritization Modules were effective, reducing the median number of variants per sample from 3222 (pre-filtering) to two (after prioritization). CNVs remaining per sample after each step of variant filtration are also plotted. **b** Distribution of the number of variants requiring manual review per sample. The majority (695/1188) of the samples had two or fewer reviewable CNVs, while nearly 95% (1109) had no more than eight reviewable CNVs. **c** SCIP further prioritized P/LP variants among reviewable CNVs, with 92.5% of them ranked first in the respective sample. CNVs were ranked by priority score and size. **d**All but two previously identified P/LP CNVs had priority scores of 1 or 2 (as determined by the SCIP Prioritization Module). While we currently select variants with priority scores < 99 for manual review, this finding indicates that further efficiencies in selecting reviewable CNVs may be possible

We next evaluated SCIP using a large collection of clinical WGS samples. We assembled this collection from two sources at The Hospital for Sick Children: a cohort of patients with cardiovascular anomalies (primarily congenital heart defects) from the Cardiac Genome Clinic (CGC, *n* = 316 families) (Reuter et al. 2020), and a cohort of patients with autism spectrum disorder from the MSSNG Project and analyzed at The Centre for Applied Genomics (TCAG) (*n* = 711 families) (Trost et al. 2022; Yuen et al. 2016), for a total of 1027 families. Because some families had multiple sequenced siblings, the 1027 families harboured 1188 non-parental WGS samples. Before filtering, they contained a median of 3,222 CNVs (inter-quartile range [IQR]: 930.75, range: 816–17,586). After the application of the Variant Filtration Module, a median of 12 CNVs per sample remained (IQR: 8, range: 1–84), reflecting > 99.5% in reduction (Fig. 6a).

The remaining CNVs were processed through the SCIP Prioritization Module, by which only a median of two CNVs per sample (IQR: 3, range: 0–40) were classified as high priority (score less than 99, Figure S3) requiring manual review. This represents an 81% further reduction (Fig. 6a). More than 13% (159/1,188) of the samples had no high priority CNVs (Fig. 6b). Taken together, the SCIP Variant Filtration and Prioritization Modules were highly efficient at CNV filtration, with < 0.1% of all CNVs requiring manual review.

### SCIP-prioritized CNVs captured all previously identified P/LP findings

We then focused on the 174 families (183 non-parental samples) with previously identified P/LP CNV findings (Figure S2). Reassuringly, all 187 previously identified P/LP CNVs were classified by SCIP as high priority for manual review, indicating that SCIP was non-inferior in sensitivity than the spreadsheet-based workflow at identifying P/LP CNVs (Materials and Methods). SCIP further prioritized the P/LP findings among reviewable CNVs: 92.5% (173/187) of them ranked first in the respective sample (Fig. 6c; *p* = 6.37 × 10^–26^, one-tailed Wilcoxon rank-sum test). In summary, SCIP was effective at prioritizing P/LP CNVs.

### SCIP had higher sensitivity than the manual workflow

Given the complexity of the manual workflow, we hypothesized that some P/LP CNVs may have remained undetected. Therefore, we re-interpreted CNVs in all 1027 families (Figure S2b) using SCIP. We identified an additional 835.20-kb pathogenic deletion at 15q11.2 (BP1–BP2) that fully contained HI region ISCA-37448, in a patient with autism spectrum disorder. This ISCA region was recurrent and previously reported in patients with autism. This result suggests that SCIP, benefitting from efficient variant filtration and user-friendly visualization, is more sensitive than the spreadsheet-based manual workflow in identifying P/LP CNVs.

### SCIP substantially reduced time burden of clinical CNV interpretation

We performed a blinded, timed, head-to-head comparison (SCIP vs. the spreadsheet-based manual workflow) of CNV interpretation with 15 ASD cases (Table S6). They were selected for good representation of CNV types and sizes (including no CNV finding) and randomized. Two analysts experienced in SCIP (1 year of experience) and the manual workflow (1.75 years of experience), respectively, blinded to case selection, was tasked with identifying reportable findings and timing the analyses using the corresponding approach. Each case was assigned different IDs for SCIP and the manual workflow.

**Fig. 7:**
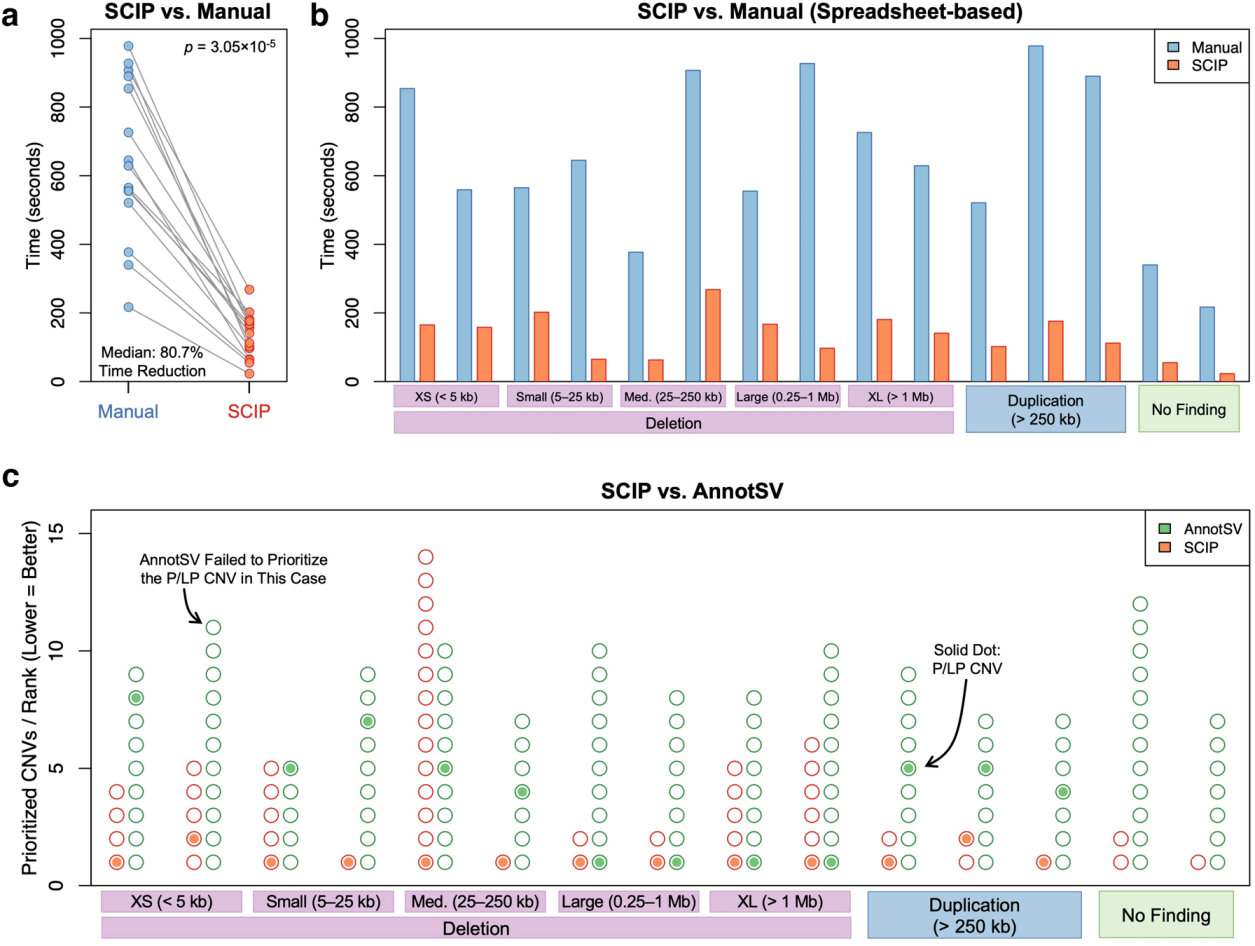
SCIP was Substantially Superior to Previous Approaches for CNV Interpretation. **a, b** In a head-to-head comparison with the spreadsheet-based manual workflow using 15 samples sequenced for autism spectrum disorder, SCIP was 80.7% (median) faster than the manual approach currently used by the CGC and TCAG at The Hospital for Sick Children. **b** The observed time savings of SCIP was consistent across a diverse range of scenarios, including deletions of varying sizes (*n* = 2 for each category), duplications (*n* = 3), and two cases with no reportable CNV findings. **c** SCIP was statistically significantly more effective at CNV prioritization than AnnotSV. Each case is represented by two columns of circles (one orange and one green). Circles indicate prioritized CNVs, while solid dots indicate P/LP CNVs. Rank of the P/LP variant among prioritized CNVs can be inferred using the Y-axis. Compared with AnnotSV, SCIP had significantly lower number of prioritized CNVs per case and better ranking for P/LP CNVs

The analysts were able to identify all reportable findings, or lack thereof, using either approach. However, SCIP was substantially faster (Fig. 7a): it took SCIP a median of 2 min 21 s (IQR: 90.5, range: 23–268 s) per case, corresponding to an 80.7%-reduction (median, IQR: 12.2, range: 64.3–90.0) in time burden compared with the manual workflow. This was statistically significant (*p* = 3.05 × 10^–5^, paired one-tailed Wilcoxon rank-sum test). In addition, SCIP was consistently faster across diverse scenarios (Fig. 7b). Thus, SCIP achieved the designed goal of substantially reducing the time burden of CNV interpretation without compromising efficacy.

### SCIP substantially outperformed AnnotSV in CNV prioritization

We then benchmarked SCIP against AnnotSV (Geoffroy et al. 2021), a recently published tool for annotation, prioritization, and tabular visualization (with the knotAnnotSV tool) of CNVs detected in clinical cases. Because AnnotSV has minimal support for variant quality assessment, we compared the performance of CNV prioritization between SCIP and AnnotSV. The 15 ASD cases selected for the above head-to-head comparison (with the spreadsheet-based workflow) were used in this analysis. Unfiltered lists of CNVs detected in these samples were provided to both SCIP and AnnotSV. We then compared the number of prioritized CNVs and rank of the P/LP variant (if any) among prioritized CNVs between the two tools.

SCIP significantly outperformed AnnotSV in both metrics (Fig. 7c). Among the 15 cases, a median of two CNVs per case were prioritized by SCIP, which was significantly less than the median of eight CNVs by AnnotSV (*p* = 8.88 × 10^–4^, paired one-tailed Wilcoxon rank-sum test). In addition, we found that in one case, AnnotSV failed to include the pathogenic variant among the prioritized CNVs. In the remaining cases, the median rank of the P/LP variant was 1 and 4.5 for SCIP and AnnotSV, respectively (*p* = 6.66 × 10^–3^, paired one-tailed Wilcoxon rank-sum test). Taken together, these findings indicate that SCIP was substantially superior to AnnotSV in prioritizing P/LP CNVs in clinical cases.

## Discussion

### SCIP is substantially better than the manual workflow and published tools

SCIP streamlines the workflow for clinical interpretation of CNVs. We rigorously evaluated SCIP using more than 1,000 families containing nearly 200 P/LP CNVs. It is noteworthy that this cohort size and the number of P/LP CNVs are almost unprecedented. Most tools were evaluated using a small number of P/LP CNVs (a few dozen or less), or only with benign or simulated variants (Belyeu et al. 2021; Minoche et al. 2021).

We revealed that SCIP was more sensitive than the spreadsheet-based manual workflow, not only selecting all previously identified P/LP CNVs for manual review, but also discovering one pathogenic CNV overlooked by previous curations. Meanwhile, CNV interpretation using SCIP was more than 60% faster per case than the manual workflow. We also showed that SCIP was significantly more effective than AnnotSV (Geoffroy et al. 2021) in CNV prioritization. These results convincingly indicate that SCIP is substantially better than previous workflows and tools.

All components of SCIP contributed to its performance. The Variant Filtration and Prioritization Modules efficiently selected < 0.1% of all variants for manual review. While the Visualization Module provided an integrated interface displaying most, if not all, information needed for CNV interpretation, keeping the back-and-forth among multiple tools to a minimum. When working in unison, fewer variants will be presented to an analyst in a more user-friendly manner, resulting in time reduction without sacrificing sensitivity.

SCIP has superior visualization capabilities compared to previously published tools, such as ClinSV (Minoche et al. 2021), samplot (Belyeu et al. 2021), CNVxplorer (Requena et al. 2021), and AnnotSV/knotAnnotSV (Geoffroy et al. 2021). Most importantly, none of these tools provide a feature analogous to Sect. 2 of the SCIP Visualization Module, i.e., summarizing evidence that may support or refute CNV quality and/or pathogenicity. The summary feature within the SCIP Visualization Module is useful in quickly showing analysts where to focus their attention during the detailed review. For example, an analyst might need to spend more time on quality assessment when alerted that the CNV was not supported by anomalous reads. Further, being a web-based tool, SCIP provides many direct links to external resources. Most other tools are unable to do so as they are not web-based. We found this feature greatly reduced time burden and error. For example, using SCIP, only one click is required to view the CNV region in the gnomAD browser, while three separate steps are needed otherwise (open the main page, enter the coordinates [error-prone], then click search). In addition, while some tools are good at variant quality assessment, and others are good at visualizing biological context, none are ideal for both. SCIP, in contrast, displays all information, including variant quality and biological context, in one unified interface.

### Limitations and future developments

Improvements may be possible in selecting CNVs for manual review. CNVs with priority scores below 99 require manual review, while nearly 99% (185/187) of the P/LP CNVs had priority score 1 or 2 (Fig. 6d). Further refinements may reduce the number of reviewable CNVs per case while maintaining sensitivity. A small subset (6.6%) of samples analyzed in this study had relatively larger numbers (> 8) of reviewable CNVs. We found that the SCIP Filtration Module had lower efficiency for these samples, the root cause of which remains to be determined. In addition, SCIP currently does not use patient phenotypes in prioritization. Incorporating patient phenotypes may further prioritize P/LP variants among the reviewable CNVs.

While most known pathogenic CNVs directly impact protein-coding genes, emerging evidence reveals that non-coding CNVs, particularly those overlapping regulatory elements, may also be causative for Mendelian disease (Flöttmann et al. 2018). SCIP currently has limited capability in analyzing non-coding CNVs. This limitation is inherent to the incomplete understanding of clinical relevance of non-coding CNVs. For well-established non-coding pathogenic regions, SCIP uses an exception list (Table S2), i.e., essentially treating them as coding. We suggest updating the exception list if new pathogenic non-coding regions are discovered in the future. Updated lists will be posted on the SCIP GitHub site. We plan to accommodate non-coding CNVs when guidelines become available in the future.

SCIP is designed for CNVs detected by WGS for constitutional genetic disorders, therefore its applicability in other scenarios may be limited (e.g., somatic CNVs in cancer or CNVs detected on exome sequencing or gene panels). Furthermore, SCIP is not intended for the visualization of full-chromosome aneuploidies. SCIP had been primarily tested in patients with congenital cardiovascular disease and/or ASD, and we plan to further evaluate SCIP in patients with other rare genetic disorders. Additionally, SCIP does not yet support the identification of compound heterozygous variants involving both CNV and SNV. This functionality will be incorporated in a forthcoming sister tool of SCIP for the clinical interpretation of SNVs.

Finally, SCIP may be unable to handle substantial CNV under-calling (identifying variants smaller than their actual sizes). This typically results from the fragmentation of a large (0.5–1 + Mb) variant, with the CNV caller identifying it as multiple discrete but nearby smaller variants. To avoid this issue, merging nearby CNVs before using SCIP is recommended (see the “Pre-processing: Merging Under-called CNVs” section in Supplementary Materials for details). We did not encounter any issue with under-calling using this approach, despite more than 50 P/LP CNVs being initially fragmented.

## Conclusions

We designed and implemented SCIP, a tool that effectively reduces the complexity and improves the efficiency of clinical CNV interpretation. SCIP was evaluated on an unparalleled cohort of more than 1000 WGS samples containing nearly 200 P/LP CNVs. SCIP had superior performance than previous workflows and tools. SCIP is fully available for implementation in clinical diagnostic laboratories, and we are confident that it will substantially improve clinical CNV interpretation.

## Supplementary Information

Below is the link to the electronic supplementary material.

Supplementary file1 (PDF 11265 KB)

## Abbreviations

ASD: Autism spectrum disorder
B/LB: Benign or likely benign
CGC: Cardiac Genome Clinic
CNV: Copy number variation
GUI: Graphical user interface
HI: Haploinsufficient/haploinsufficiency
IGV: Integrative Genomics Viewer
OMIM: Online Mendelian Inheritance in Man database
pext: Proportion expressed across transcripts
pLI: Probability of being loss-of-function intolerant
P/LP: Pathogenic or likely pathogenic
popmax: The continental population with the highest allele frequency (gnomAD)
RAM: Random access memory
SCIP: Suite for CNV Interpretation and Prioritization
SNV: Single-nucleotide variants
SV: Structural variation
TCAG: The Centre for Applied Genomics
TS: Triplosensitive/triplosensitivity
WGS: Whole-genome sequencing
