# Supplementary material for: SCIP: software for efficient clinical interpretation of copy number variants detected by whole-genome sequencing
Source: Hum Genet. 2022 Nov 14;142(2):201–16. doi: 10.1007/s00439-022-02494-1 (PMC9918589; doi:10.1007/s00439-022-02494-1)
Supplement: Supplementary file 1 — Supplementary file1 (PDF 11265 KB) [file 439_2022_2494_MOESM1_ESM.pdf]

Supplementary Materials

for

SCIP: software for efficient clinical interpretation of copy-number variants detected by whole-genome sequencing

Contents

Supplementary Methods
Supplementary Text 1–3
Supplementary Figures 1–10
Supplementary Tables 1–6
Supplementary References

Supplementary Methods

Files Generated In-house Used by SCIP

gnomad_v2.1_sv.sites_hg38.vcf.gz*.* To generate this file, POS and END positions in the hg19 gnomAD-SV sites VCF file (gnomad_v2.1_sv.sites.vcf.gz) were converted to hg38 coordinates using liftOver (default settings). END2 and POS2 coordinates were ignored. The hg38 VCF file was generated using the converted coordinates, and only contains POS, END, SVTYPE and POPMAX_AF information. SVs were not included in this new file if their sizes in hg38 were less than 80% or more than 120% of that in hg19. This file was then compressed using bgzip and indexed using tabix.

gnomAD.pext.bed.gz (hg19) and gnomAD.pext.hg38.bed.gz (hg38). The gnomAD base-level pext TSV file (all.baselevel.021620.tsv.bgz) was processed to generate the hg19 file by taking the maximum pext score by position across tissues. liftOver with default settings was then used to generate the hg38 version. The BED files were compressed using bgzip and indexed using tabix.

gnomADv2.1_commonCNV.gene.txt (hg19) and gnomADv2.1_commonCNV_hg38.gene.txt (hg38). Deletions and duplications with POPMAX_AF above 1% and the PASS flag in the FILTER column were extracted from the gnomAD-SV sites VCF file. Protein-coding genes fully contained within these CNVs were then identified (gene information from the BioMart tool of the Ensembl Genome Browser (Howe et al. 2021)).

hg19_ClinVar_CNV_20211020.txt (hg19) and hg38_ClinVar_CNV_20211020.txt (hg38). ClinVar pathogenic and non-pathogenic CNVs, in BED format, were downloaded from https://ftp.ncbi.nlm.nih.gov/pub/dbVar/sandbox/dbvarhub/. Copy number loss (gain) was considered as synonymous with deletion (duplication), respectively. Protein-coding genes that partly or fully overlap with the CNVs were then identified (gene information from the BioMart tool of the Ensembl Genome Browser (Howe et al. 2021)).

gencc_09152021_tab_sep.txt*.* The “Submissions (tsv)” file was downloaded from the GenCC website: https://search.thegencc.org/download and converted to tab-separated using R:

```
x=read.table ("gencc-submissions.tsv",sep=",",head=T)
write.table(x,file="gencc_09152021_tab_sep.txt",quote=F,col.names=F,row.names=F,sep="\t")
```

hg19_coding_exons_v3_name_range.txt (hg19) and hg38_coding_exons_v3_name_range.txt (hg38). Exon information, including coordinates, gene name, type, and start/end phase, was obtained from the Ensembl BioMart. Non-coding exons were excluded with limited exceptions (Table S2). We also excluded exons that were not annotated as polymorphic pseudogenes or protein-coding. If exons were partially coding (i.e., the first or last coding exon flanked by UTRs), the non-coding parts were excluded. Finally, to cover canonical splice sites, the exon

coordinates were extended 2 bp at intron-exon junctions. Exons of different transcripts of the same gene were then merged for gene-level output.

`hg19_coding_exons_plotting_v2.txt` (hg19) and `hg38_coding_exons_plotting_v2.txt` (hg38). The same approach in generating the `hg19/38_coding_exons_v3_name_range.txt` files was used, with two differences. First, exons were not extended for 2 bp at intron-exon junctions, as we intend to plot exon boundaries exactly. Second, exons fully contained within another exon of the same gene (caused by alternative splicing) were discarded, as they do not contribute additional information for plotting.

`hg19_exon_jct.txt` (hg19) and `hg38_exon_jct.txt` (hg38). Coordinates from all exons (no additional filtering) were obtained from the Ensembl BioMart and re-formatted.

`hg19_GTEx_MANE_selected_transcripts_info.txt` (hg19) and `hg38_GTEx_MANE_selected_transcripts_info.txt` (hg38). We obtained transcript-level expression data from the GTEx Project (GTEx Consortium 2017) (v8) and then calculated the median expression level of each transcript across all tissues. Transcripts that were not annotated as polymorphic pseudogenes or protein-coding were excluded. For each gene, we ordered transcripts in descending order by median expression level, and sequentially selected biologically relevant transcript(s) as follows. The 1st transcript was always selected, then for the nth transcript, it was selected if its expression level was at least 20% of that of the (n-1)-ranked transcript and at least 10% of that of the 1st transcript. Selection was terminated if at least one of the two conditions was not met. We then added the MANE Select, MANE Plus Clinical, and canonical (hg38) transcripts as biologically relevant transcripts (in case they were not already selected based on expression). For these transcripts, we obtained exon count, coordinates, and coding information from the Ensembl BioMart. There are six tab-separated columns in this file: (1) Ensembl gene ID, (2) Ensembl transcript IDs for the biologically relevant transcripts (space-separated), (3) number of exons in each transcript (space-separated), (4) coding exon coordinates by transcript (space-separated between transcripts), (5) coordinates of all exons by transcript (space-separated between transcripts), and (6) whether MANE Select, MANE Plus Clinical or canonical in hg38 (space-separated).

CNV Quality Score

We computed a quality score for each CNV, displayed in section 2 of the Visualization Module (Figure 2c). The score has three discrete levels: manual (default), passed, and failed. We calculated depth ratio as the median read depth within vs. flanking the variant (50% of the CNV size or 100 kb, whichever is greater, on both sides). In other words, an ideal heterozygous deletion or duplication will have a depth ratio of 0.5 or 1.5, respectively (Figure S1).

The default score is manual, i.e., unable to classify. If a deletion has depth ratio $\leq$ 0.1, it will be classified as possibly homozygous requiring manual review. If a CNV has at least two opposing anomalous reads, i.e., supporting the opposite CNV type, it will be marked as a

possible complex CNV. Otherwise, a CNV that has a median mapping quality within the CNV ≥ 50 and meets one of the following criteria will be classified as passed: (1) deletion with depth ratio ≤ 0.75 or duplication with depth ratio ≥ 1.25, and ≥ 2 supporting anomalous reads, or (2) size ≥ 10 kb, and deletion with depth ratio ≤ 0.7 or duplication with depth ratio ≥ 1.3. A CNV will be classified as failed if size ≥ 2 kb, median mapping quality ≤ 40, and has no supporting anomalous reads. While this scoring system has the potential to be improved as a hard filter, it currently is intended as guidance only. For CNVs classified as failed, manual quality review is encouraged before excluding them.

Recurrence Regions and Recurrent Breakpoints

In this study, the largely unrelated and unaffected parents of the probands were used to identify recurrence regions and breakpoints, which contain population variants and recurrent false positives. This step is intended to complement population-based filtering, since gnomAD-SV does not contain recurrent false positive CNV calls. Removing recurrent false positives is essential in minimizing CNVs requiring manual review. For hg19 and hg38, 506 and 952 parental samples were used, respectively. Recurrence regions and breakpoints were identified for deletions and duplications separately. Because breakpoint based CNV calls (i.e., from Manta) were available for the hg19 samples only, we did not generate recurrent breakpoints for hg38.

For recurrence regions, we identified genomic positions that overlap at least n_1 parental deletions (duplications), unless they were within dosage sensitive regions (score = 1–3): in this case they must overlap at least n_2 parental deletions (duplications). We intended n_2 to be more conservative – n_1 was set as 5 (both hg19 and hg38), while n_2 was set as 30 (hg19) or 50 (hg38), respectively. Adjacent positions were then merged into intervals using `bedtools`. In total, 126.05 Mb (hg38, deletion), 137.18 Mb (hg38, duplication), 59.08 Mb (hg19, deletion), and 62.33 Mb (hg19, duplication) of the genome were identified as recurrent, respectively. We generated a low-quality region file (i.e., `lowqual_reg` in Table S3) by adding ClinGen dosage sensitivity unlikely (score = 40) regions, genome gaps, centromeres, segmental duplications, and simple repeats.

We used hierarchal clustering to identify recurrent breakpoints. For each chromosome, a distance matrix was calculated based on breakpoints of deletions (duplications) in all samples (using the R function `dist`), followed by clustering using the `hclust` function. The tree was then cut at 2,500 using the `cutree` function, and clusters with at least five elements were retained as recurrent breakpoints. We extended five standard deviations from the mean (both sides) as the range of each recurrent breakpoint.

Recurrence regions and breakpoints contain regions/breakpoints prone to false positive CNV calls, which may differ among sequencing technologies and bioinformatics workflows. Thus, SCIP users are encouraged to identify their own recurrence regions and breakpoints if a

sufficiently large (e.g., at least 300–500 unrelated and unaffected samples) internal cohort is available.

Pre-processing: Merging Under-called CNVs

As mentioned in the Discussion, SCIP is sensitive to substantial under-calling of CNVs. This is usually caused by fragmentation of a large CNV by genome gaps or other low-quality regions, resulting in multiple small but nearby variants. Specifically, the read depth plot in the SCIP Visualization Module uses CNV-flanking regions for normalization between the clinical and reference samples. No issues are expected if the actual CNV is less than 100 kb or 50% larger (whichever is greater). However, if the actual variant is more than 200 kb or 100% larger (whichever is greater), false normalization may cause the plot to show identical read depth in the two samples and no change at the boundaries. This may result in the incorrect exclusion of a true CNV.

This issue is easily resolvable by adding a pre-processing step to merge these CNVs before providing them to the SCIP Variant Filtration Module. In this study, we first merged CNVs in the same direction (e.g., all deletions) and were > 10 kb in size, when they were < 250 kb apart, using the `bedtools` (Quinlan and Hall 2010) merge function (`-d 250000`). We subsequently de-merged regions that had < 70% identified as CNV before merging. Because SCIP handles over-calling (i.e., erroneously calling nearby discrete CNVs as one) easily, the 250 kb distance threshold was chosen to merge most, if not all, under-called CNV fragments, while accepting small risk of merging discrete nearby CNVs. The 70% CNV content threshold further reduces risk of merging discrete CNVs. Using actual data, this approach was found effective in merging fragmented CNVs while mostly avoided merging truly discrete variants. With this pre-processing, we had no issue with under-calling, despite that a substantial number of P/LP CNVs were initially fragmented.

Supplementary Text 1

SCIP Usage Instructions

(Latest version available at https://github.com/qd29/SCIP)

When using SCIP for the first time, please follow Supplementary Text 2 for initial setup steps. This instruction assumes that all setup steps have been successfully completed. We use `./` to denote the directory created for SCIP in Supplementary Text 2.

Prerequisites:

Filtration & Prioritization Modules: (a) A UNIX-based operating system (tested with CentOS 7). (b) The following software in the `$PATH` environment variable (version tested in parenthesis): `Perl` (v5.16), `R` (v3.5.1), `samtools` (v1.10), `bedtools` (v2.26), and `tabix` (v0.2.5).

Visualization Module: Any operation system supported by RStudio and has a web browser (tested with Windows 10 and macOS Big Sur).

Prepare Input Files (Variant Filtration & Prioritization Modules)

1. For each sample, prepare a tab-delimited file named `[name].unfiltered_CNV.txt` that includes all CNVs. Place the file in `./user_data`.
    a. This file has 7 columns. The first four columns are chromosome, start position, end position, and type (DEL/DUP), respectively. The fifth and sixth columns are not used by SCIP and may contain free text. The last column is sample ID.

       Example format:

       ```
       1 620001 635000 DUP . . SAM-001-001
       ```
    b. To allow SCIP to recognize members of the same family, we recommend naming samples using the following format: ABC-123-001. ABC-123 is the family ID that is identical for all family members. The last three digits denote family relationship, with 001, 002, 003, 004+ denoting proband, mother, father, and additional family members, respectively.
2. In the Filtration & Prioritization Modules Configuration File (`pipeline_config.txt` (hg19) or `pipeline_config_hg38.txt`), specify `SAMPLE_ID` as the path to the file containing conversion information between sample ID and alignment file name. If this file already exists, you may append information about the current sample to this file.
    a. Two tab-separated columns. Each row is a sample. The second column is the sample ID; the first column is the name prefix of the alignment file for this sample.
    b. For example, if alignment file for sample `SAM-001-001` is `alignment001.bam`, specify the following line in this file:

```
alignment001 SAM-001-001
```

   c. Example file available at `./hg[19/38]_files/demo/sample_id.txt`.

3. In the Filtration & Prioritization Modules Configuration File (`pipeline_config.txt` (hg19) or `pipeline_config_hg38.txt`), specify `cohort_CNV` as the path to the file containing CNVs detected in internal cohorts. If this file already exists, you may append information about the current family to this file. This file is optional, however, if one would like to study transmission pattern, CNVs from family members must be included in this file.
   a. Optional information. If a user does not wish to provide this file, specify the path to an empty file.
   b. Six tab-separated columns. Each row is a CNV. The first four columns are chromosome, start position, end position, and type (DEL/DUP), respectively. The fifth column is sample ID, and the last column is the algorithm used to identify this variant. For example:

```
1 10001 50001 DUP SAM-001-001 ERDS
```

Run the SCIP Variant Filtration & Prioritization Modules

4. Run the following command. Denote the `[name]` specified in step 1 with the `-n` flag. For example (for the script name, change `hg19` to `hg38` as appropriate):

```
perl SCIP_backend_hg19.pl -n SAM-001-001
```

   a. Expected outputs on screen. Filtration Module: the following information will be printed on screen - `SCIP Filtration Module script 01/02/03 processing hg19/hg38 chr[1-22,X]`. Prioritization Module: the current date/time, name of the CNV being analyzed, and whether it generates new / reuses SAM and depth files will be printed.
   b. `samtools` and `tabix` may occasionally report warnings, e.g., the index file is older than the data file, protocol not supported, and/or failed to open reference (especially for CRAM files). No other errors/warnings are anticipated.

5. **(Optional, Advanced Users Only)** Clean-up. SCIP stores SAM and depth information extracted from alignment files in the `./d1temp_server` directory. These files are no longer required after step 4 and may be removed.
   a. If the CNVs are re-analyzed in the future (e.g., using up-to-date annotation files), keeping these files allow SCIP to use them instead of querying the alignment BAM/CRAM files again. Therefore, we recommend keeping them (unless disk space poses an issue).

Use the SCIP Visualization Module

6. Modify the `LIST_NAME` entry in the `interface_config.txt` to `[name].[hg19/hg38]` (see step 1).
7. Open `SCIP_interface.R` (or `SCIP_interface_hg38.R`) in RStudio. Click “Run App”, then at the top of the pop-up window, click “Open in Browser”. We recommend using Google Chrome.
    a. RStudio sometimes produces a warning, which can be discarded unless it is a fatal error (e.g., the Visualization Module crashes).

CNVs are now ready for manual review using the SCIP Visualization Interface.

Supplementary Text 2

SCIP Setup Instructions

(Latest version available at https://github.com/qd29/SCIP)

<u>Filtration & Prioritization Modules</u>

Prerequisites: (a) A UNIX-based operating system (tested with CentOS 7). (b) The following software in the `$PATH` environment variable (version tested in parenthesis): `Perl` (v5.16), `R` (v3.5.1), `samtools` (v1.10), `bedtools` (v2.26), and `tabix` (v0.2.5).

1. Download the required annotation file at the following links. Place the file in a folder you intend to install the SCIP backend modules.

   https://drive.google.com/file/d/1rWwkJ-eFDL1xT0DzraNi9oQ-NtC62TCe/view (hg19)
   https://drive.google.com/file/d/1N4dt_UZ3CMw7CtkmU-KQDZe2tFmnt9kd/view (hg38)

2. At the command line, copy the following code block (depending on the genome build) and press the enter/return key to execute. The SCIP Filtration & Prioritization Modules will be installed in a new folder called `SCIP_backend`. For simplicity, this folder will be hereafter referred to as `./`.

   For hg19:

```
mkdir -p ./SCIP_backend
mv ./SCIP_hg19_files.tar.gz ./SCIP_backend
cd ./SCIP_backend
tar -xvzf ./SCIP_hg19_files.tar.gz
mkdir -p d1temp_server d1stat user_data app_temp_file
git clone https://github.com/qd29/SCIP.git
mv ./SCIP/filtration_prioritization_hg19/* ./
rm -rf ./SCIP
```

   For hg38:

```
mkdir -p ./SCIP_backend
mv ./SCIP_hg38_files.tar.gz ./SCIP_backend
cd ./SCIP_backend
tar -xvzf ./SCIP_hg38_files.tar.gz
mkdir -p d1temp_server d1stat user_data app_temp_file
git clone https://github.com/qd29/SCIP.git
mv ./SCIP/filtration_prioritization_hg38/* ./
rm -rf ./SCIP
```

3. Obtain the OMIM `genemap2.txt` file from https://www.omim.org/downloads, registration required. Place the file in the `hg19_files` or `hg38_files` folder under `./`.

Filtration & Prioritization Modules Configuration File

Configuration File Name: `pipeline_config.txt` (hg19) or `pipeline_config_hg38.txt`

4. Specify `ALIGNMENT_PATH` to the path containing alignment files. Each sample must have its own subdirectory. For example, alignment file for sample001 should be stored at:

   ```
   ALIGNMENT_PATH/sample001/sample001.bam
   ```

   Corresponding index files (`bai` or `crai`) must also be available.

5. Specify `REF_BAM` as the path to the whole-genome alignment file of a reference sample.

   `NA12878_S1.bam`: ftp://ftp.sra.ebi.ac.uk/vol1/run/ERR194/ERR194147 (hg19)
   `NA12878.final.cram`: ftp://ftp.sra.ebi.ac.uk/vol1/run/ERR323/ERR3239334 (hg38)

   a. Corresponding index files (`bai` or `crai`) must also be available. For the hg19 BAM file, EBI did not provide an index, thus you need to generate the index with the `samtools index` command.

   b. For CRAM files, you may need to convert it to BAM and/or set the `$REF_PATH` and `$REF_CACHE` environment variables, so that reads can be printed using the `samtools view [file]` command.

Setup of the SCIP Filtration & Prioritization Modules is now complete. These steps are required only once. To run the Filtration & Prioritization Modules, go to step 1, Supplementary Text 1.

Advanced Users: (1) Consider periodically update the annotation files (e.g., OMIM, GenCC, ClinGen). (2) The `expression_file`, `GO_terms`, `gene_interest`, and `search_terms` entries in the configuration file can be customized by the user. See Table S3 for formatting details.

Visualization Module

6. The SCIP Visualization Module can be run on Windows or macOS computers. Create an empty directory on the computer (hereafter referred to as the working directory).

7. Download the `SCIP_interface.R` (hg19) or `SCIP_interface_hg38.R` script and the `interface_config.txt` files from GitHub, place them in the working directory.

8. Install `R`, `RStudio`, and R packages `shiny`, `DT` and `plotrix` (and any dependencies).

9. The `./` directory of the Filtration & Prioritization Modules needs to be accessible to the computer running the Visualization Module. At our institution, this was done by

mounting the computer server running the Filtration & Prioritization Modules as a network volume on the PC running the Visualization Module.

   a. When the network volume option is not available, the user can download all files in the `./app_temp_file` and `./user_data` directories to a local directory. Note that the relative location of `app_temp_file` and `user_data` must be maintained (i.e., they must sub-directories within the same directory).

10. In the `interface_config.txt` file:
    a. Modify the `TEMP_FILE_DIR` entry to the path to the `app_temp_file`.
    b. Modify the `ROOT_DIR` entry to the path to the working directory (see step 6).
    c. Modify the `USER` entry with your identifier (e.g., name/email). This is optional and is only used to track interpretations across multiple users.

Setup of the SCIP Visualization Module is now complete. These steps are required only once. **Exception:** if you use the approach described in step 9a, you will need to re-download the `./app_temp_file` and `./user_data` directories every time you have new samples. To run the Visualization Module, go to step 6, Supplementary Text 1.

Supplementary Text 3

Typical Use Cases of SCIP

Scenario 1: reportable pathogenic deletion. MSG-2959-001 (also known as AU2289301) is a patient with autism spectrum disorder. The parent-offspring trio was sequenced. Only one CNV was classified by SCIP as high priority for manual review. Section 2 of the Visualization Module highlighted that this deletion, well-supported by anomalous reads, overlapped *CHD2*. This gene is curated and predicted to be HI, is an autism candidate gene, and is associated with an autosomal dominant neurodevelopmental disorder (Figure 2c). We concluded that this CNV is of good quality after reviewing sections 3 and 4 (Figure 3). In section 5 (Figure 4), no gnomAD-SV population variation and no ClinVar benign/likely benign (B/LB) variant were found overlapping *CHD2*. This variant was not found in other members of the MSG-2959 family, indicating *de novo* occurrence. In section 6 (Figure 5), we found that this CNV removed exons 1–7 (of 39) of the biologically relevant transcript of *CHD2*. ClinGen dosage curation confirmed that *CHD2* HI was reported in patients with autism and intellectual disability. Thus, this CNV was interpreted as pathogenic per the ACMG/ClinGen guidelines (codes: 2C-1, 4C).

Scenario 2: reportable pathogenic duplication. MSG-3175-001 (also known as AU3160302) is a patient with autism spectrum disorder. Here we describe the manual review of the first of two CNVs that were classified as high priority by SCIP. Section 2 of the Visualization Module highlighted that this 736-kb duplication at 16p11.2 fully contained a ClinGen TS region (Figure S8a). A review of section 3 indicated that this duplication is of satisfactory quality (Figure S8b). Although there were no anomalous reads supporting this variant, this is acceptable for larger CNVs. With Section 6 (and link to ClinGen dosage map), we confirmed that ISCA-37400 was fully contained in this CNV, and TS features include autism (Figure S8c). This CNV was thus interpreted as pathogenic (code: 2A).

[The second variant that was classified as high priority was a pathogenic 966-bp homozygous intragenic deletion of *CLN3*. This variant had a priority score of 5, because *CLN3* is associated with autosomal recessive ceroid lipofuscinosis, neuronal, 3 (MIM 204200, also known as the Batten disease). This variant has identical gene content as the 1.02-kb deletion responsible for 73% of Batten disease patients (The International Batten Disease Consortium 1995). While autism is not a reported feature of Batten disease, the patient had scored very low (score = 56) on the Global Ability Composite Estimate, indicating profound impairment in intellectual ability, verbal and nonverbal ability, and/or motor and language skills. One feature of Batten disease is psychomotor degeneration, and it is plausible that both the 16p11.2 duplication and the *CLN3* intragenic deletion contributed to the phenotypes of the patient. Due to the limited clinical information available, we were unable to confirm whether this patient has vision loss, which is the most prominent feature of Batten disease.]

Scenario 3: population variation. HSC-200-001 is a patient with congenital heart defects. An 85-kb duplication was selected for manual review. While the quality of this duplication was satisfactory, the SCIP Internal and External Variant Databases section (Figure S9) revealed

that it was a population variation. More than ten similar-sized duplications were also reported as B/LB in ClinVar. Using links in the SCIP table, we confirmed that duplication of this region was found in 48 gnomAD-SV samples. This variant was ruled out as pathogenic due to being a population variant.

Scenario 4: not affecting a biologically relevant transcript. HSC-213-001 is a patient with congenital heart defects and neurodevelopmental disorders. A 1.7-kb deletion was selected for manual review. This deletion was of good quality. However, section 2 of the Visualization Module indicated that the maximum pext score within this CNV was zero (Figure S10a), indicating that no exons within this deletion were found expressed in any GTEx tissue (GTEx Consortium 2017). Upon review of section 6, we confirmed that the only exon affected by the CNV was not on the MANE Select transcript and had zero pext, while nearby exons had high pext scores (Figure S10b). This variant was ruled out since it did not impact a biologically relevant transcript.

Supplementary Figures

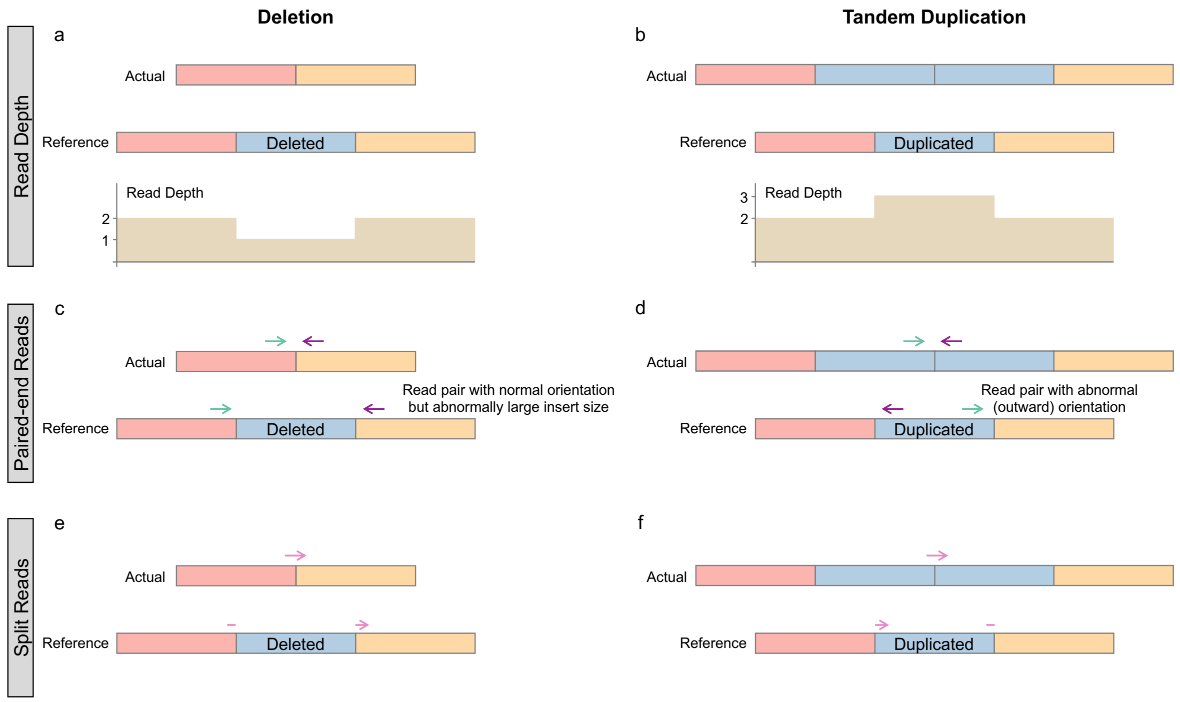

Figure S1. Genomic Signatures of Deletions and Tandem Duplications

The deleted (panels a, c, e) or duplicated (panels b, d, f) genomic segment was coloured in blue. There are three signatures for deletions or tandem duplications when reads were mapped to a reference genome that does not contain the CNV. (a, b) Read depth. The read depth at the deleted or duplicated region is expected to decrease (panel a) or increase (panel b), respectively. (c, d) Paired-end reads with abnormal insert size and/or orientation. The green and purple arrows denote the two reads in a read pair. In the actual genome (top plots), the two reads always have an inward orientation and the distance between the two reads (i.e., insert size) approximately follows a pre-determined value. When mapped to the reference genome, the read pair is expected to show an abnormally large insert size with correct orientation (for deletions, panel c) or an abnormal (outward) orientation (for duplications, panel d). (e, f) Split reads. Reads that span the deletion or duplication junction (magenta arrows) are expected to show a “split” pattern when mapped to the reference genome, i.e., for a given read, part of it mapped to one location, while the other part mapped to another location far away.

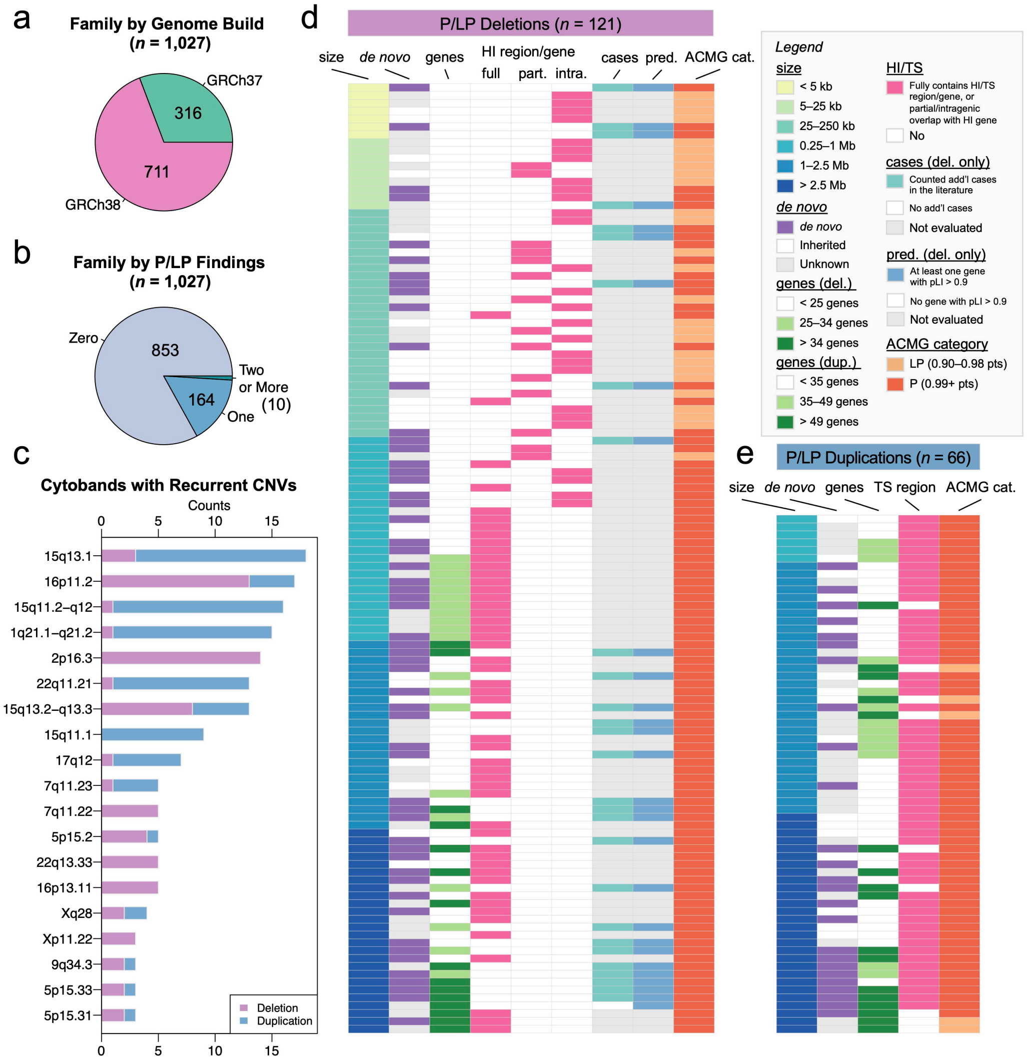

Figure S2. A Diverse Cohort of Families and CNVs for Comprehensive Evaluation of SCIP

Families by (a) genome build used for alignment, and (b) P/LP findings. A total of 183 non-parental samples from 174 families had at least one P/LP CNV (totaling 187). (c) Cytobands that harbour at least three P/LP CNVs. (d, e) Details on the P/LP deletions (d) and duplications (e). The HI region/gene columns in panel d indicate that the deletion fully contains a HI region (full), partially overlaps a HI gene (part.) or is intragenic within a HI gene (intra.), respectively. The TS region column in panel e indicates that the duplication fully contains a TS region. All P/LP CNVs scored at least 0.9 points (LP threshold) using the ACMG/ClinGen guidelines.

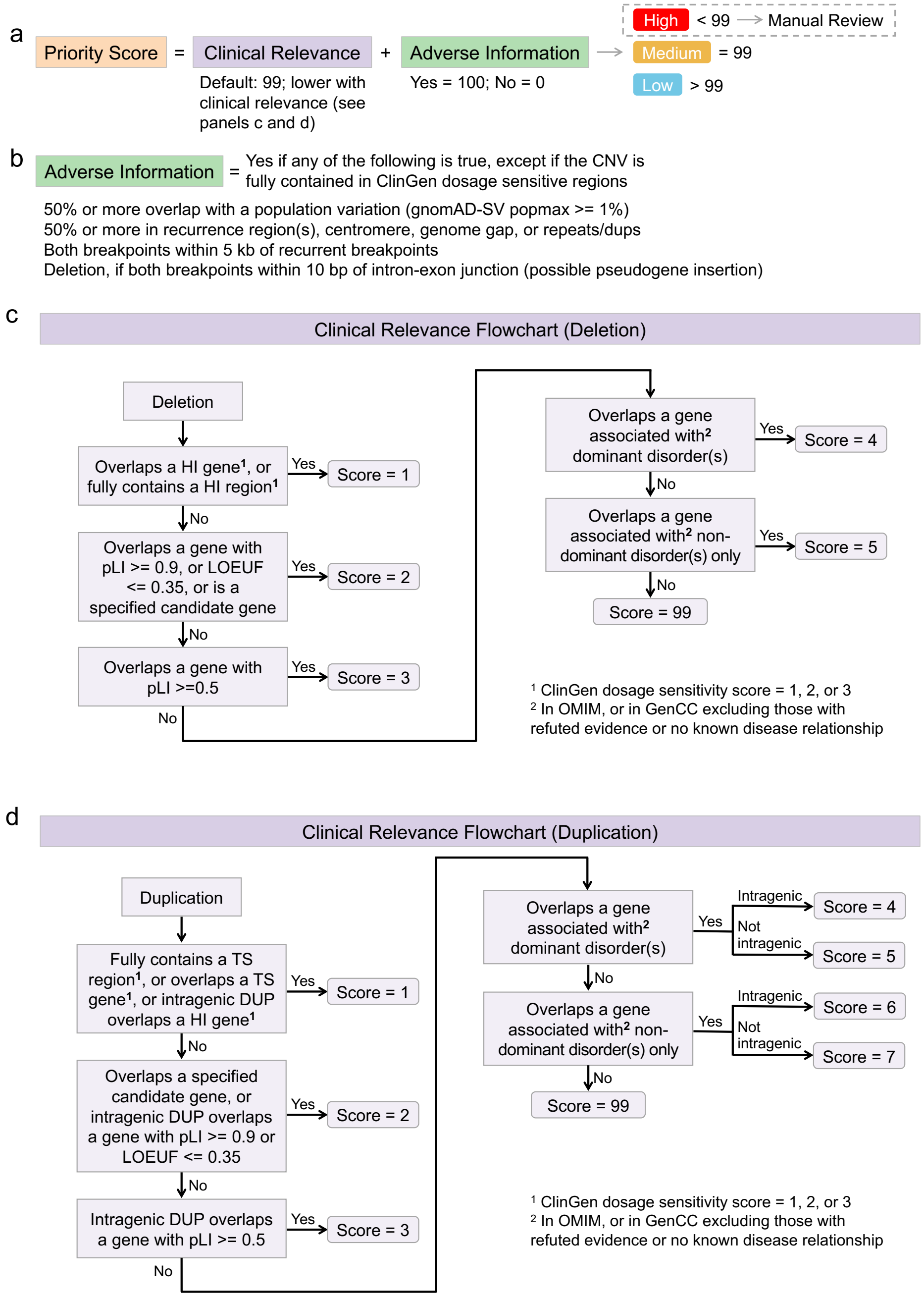

Figure S3. Details on the SCIP Prioritization Algorithm

(a) The priority score determined by the SCIP Prioritization Module is the sum of two scores: clinical relevance and adverse information. Based on the score, a variant can be classified as of high, moderate, or low priority. Only CNVs with high priority require manual review. (b) Method for determining the adverse information score. (c and d) Flowcharts used to assign the clinical relevance score for deletions (c) and duplications (d).

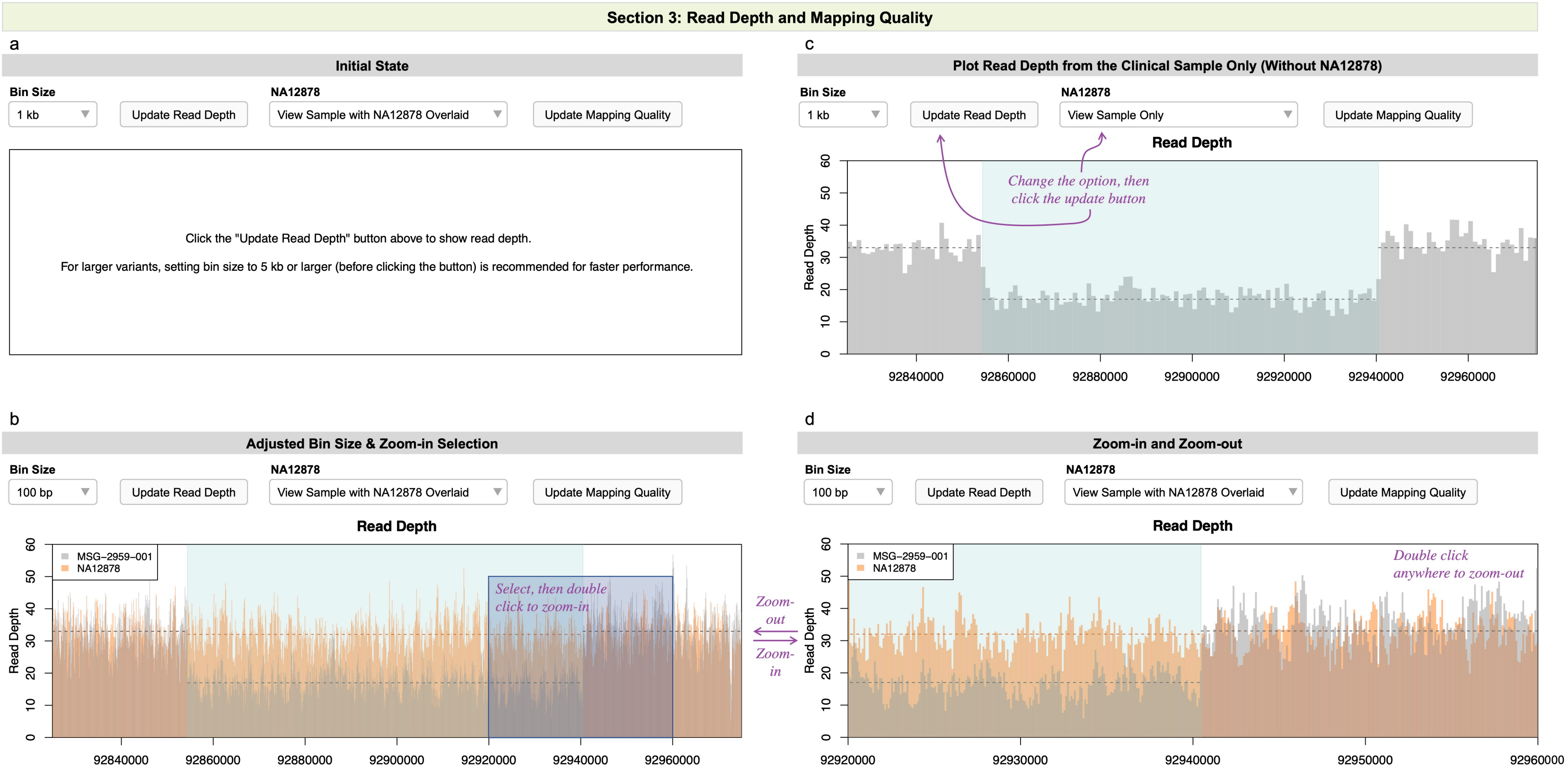

Figure S4. Details on the Read Depth and Mapping Quality Section of the SCIP Visualization Module

While only the Read Depth panel is shown, the illustrations are also applicable to the Mapping Quality panel. Comments are shown in purple. (a) Initial state of the Read Depth and Mapping Quality section. Click the “Update Read Depth” and “Update Mapping Quality” buttons to display the plots. (b) Same plot as in Fig. 3a, with smaller bin size (100 bp). Bin size can be adjusted using the drop-down menu, then by clicking the update buttons. All plots in the Module are fully interactive, allowing zoom-ins/outs. To zoom-in, select a region in the plot (shaded dark blue rectangle) and double click. All plots are automatically updated with the selected genomic interval. See panel d for a zoomed-in view. (c) Same plot as Fig. 3a, plotting data from the clinical sample only. Select “View Sample Only” in the NA12878 drop-down menu, then click the update buttons to toggle this plot. (d) Zoomed-in view of panel b. To zoom out, double-click anywhere in the plot.

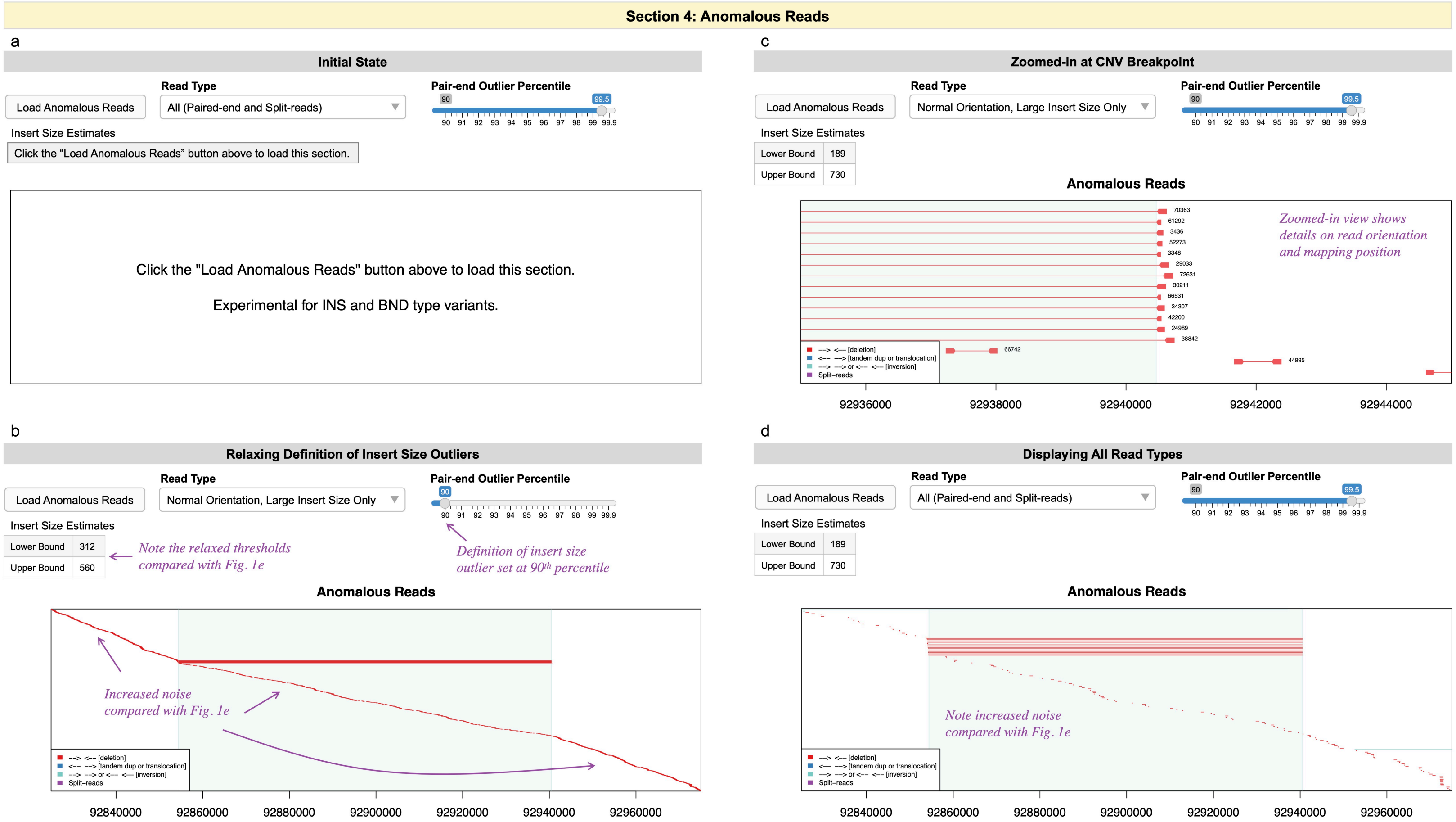

Figure S5. Details on the Anomalous Reads Section of the SCIP Visualization Interface

Comments are shown in purple. (a) Initial state of the Anomalous Reads section. Click the “Load Anomalous Reads” button to display the plot and tables. (b) Same plot as in Fig. 3b, with relaxed definition of insert size outliers (the slider at 90th percentile) causing increased noise (more read pairs irrelevant to the CNV) in the plot. (c) Zoomed-in plot at the 3’-breakpoint of the CNV, showing finer details on read orientation (arrow) and mapping position. (d) Same region as Fig. 3b but displaying all read types (adjustable using the “Read Type” drop-down menu), resulting in increased noise.

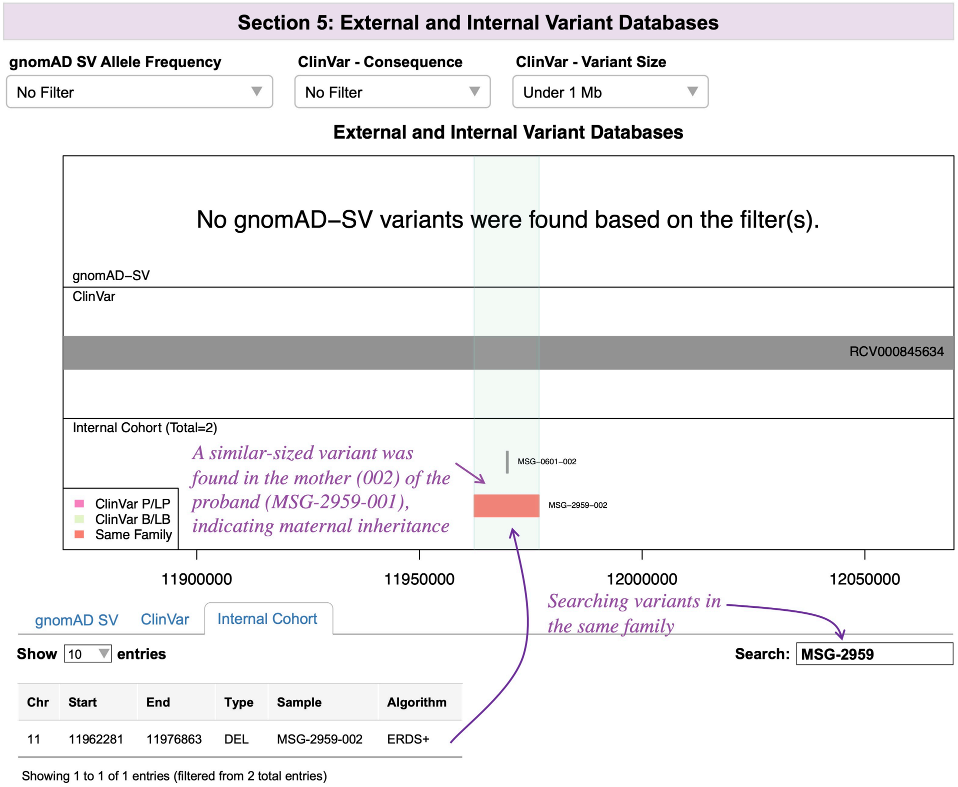

Figure S6. External and Internal Variant Databases Section for an Inherited Variant

This figure shows the External and Internal Variant Databases section of the SCIP Visualization Interface, for a deletion detected in the proband of MSG-2959 family. The deletion region (green shade) does not overlap any gnomAD-SV population variation (top panel) or any ClinVar P/LP or B/LB variants (middle panel). In the bottom panel, there is a similar-sized deletion found in MSG-2959-002 (002 denotes the mom of the proband). This deletion is coloured in red because it was found in the same family. This information is confirmed by searching the Internal Cohort table. Therefore, it can be concluded that this deletion in the proband is maternally inherited. In the figure, comments are shown in purple.

Section 6: Genomic Neighbourhood

a

Genes | ClinGen Dosage Map

Show 10 **entries** **Search:**

**Name**	**Chr**	**Start**	**End**	**Haploinsufficiency**
CHD2	15	92900324	93027996	Sufficient (3)

Showing 1 to 1 of 1 entries

b

Genes | ClinGen Dosage Map

Show 10 **entries** **Search:**

**Name**	**Chr**	**Start**	**End**	**Haploinsufficiency**	**Triplosensitivity**
ISCA-37446	22	18924718	21111383	Sufficient (3)	Sufficient (3)
ISCA-37433	22	18924718	20299685	Sufficient (3)	Sufficient (3)
ISCA-37516	22	20377696	21111383	Emerging (2)	Little (1)
TBX1	22	19756703	19783593	Emerging (2)	No evidence (0)
CRKL	22	20917407	20953747	Little (1)	No evidence (0)
GP1BB	22	19723539	19724771	Recessive (30)	
SCARF2	22	20424584	20437825	Recessive (30)	No evidence (0)
SLC25A1	22	19175581	19178736	Recessive (30)	

Showing 1 to 9 of 9 entries

Figure S7. The ClinGen Dosage Map Table in the SCIP Visualization Interface

(a) For a deletion that overlaps a ClinGen HI gene (the same CNV as shown in Figures 2–5). (b) For a duplication that fully contains a ClinGen TS region. Note that the triplosensitivity column is only displayed for duplications.

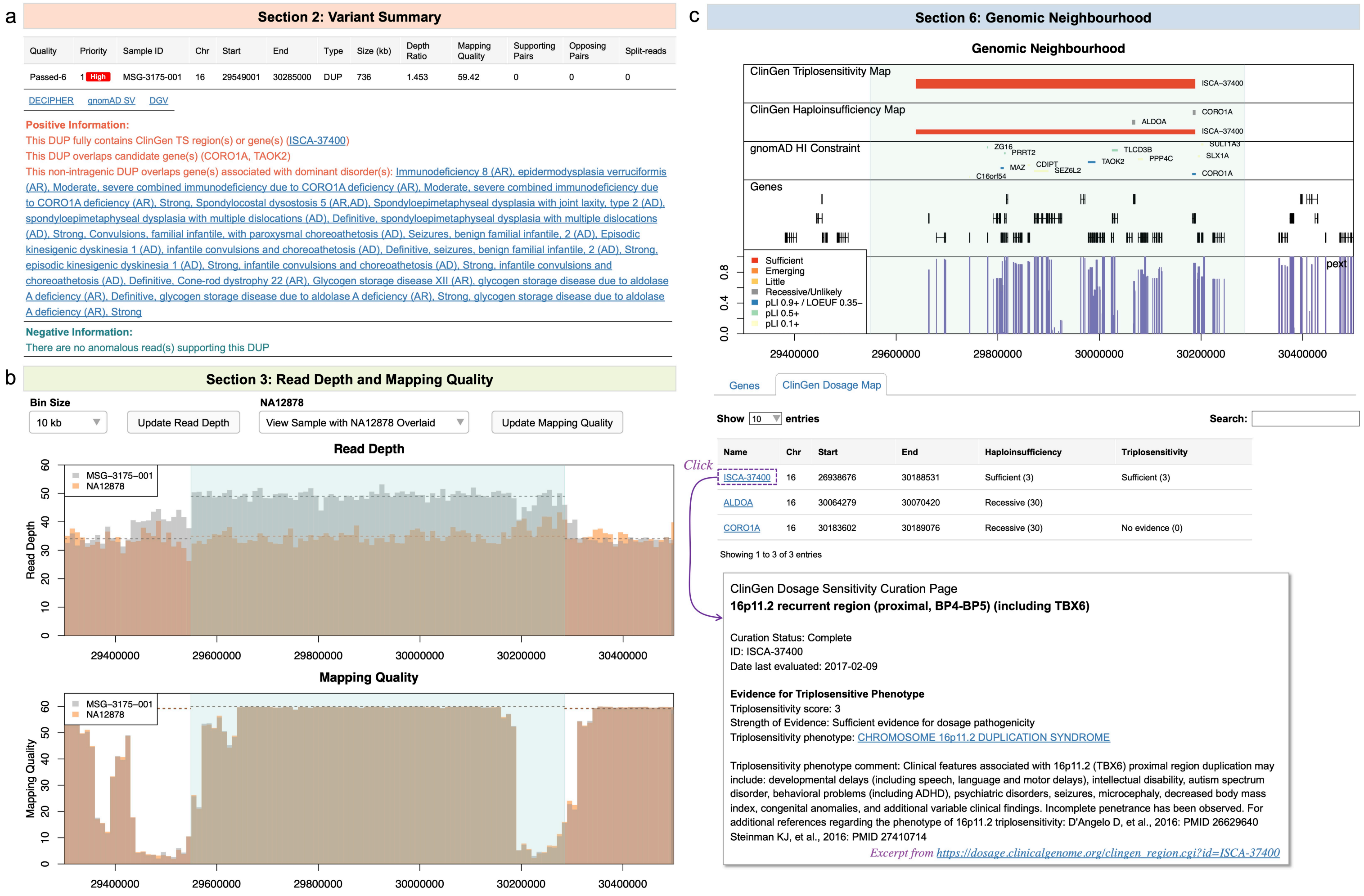

Figure S8. Use Case Scenario 2: a Reportable Pathogenic Duplication

This figure shows selected sections of the SCIP Visualization Module for a 16p11.2 duplication in a patient with autism spectrum disorder. (a) The Variant Summary section reveals that this duplication fully contains a triplosensitive (TS) region. (b) The Read Depth and Mapping Quality section shows that this CNV is of satisfactory quality. (c) The Genomic Neighbourhood section visually demonstrates that this duplication fully contains ISCA-37400, and a link to the ClinGen dosage map confirms that TS features of this region include autism. Comments are in purple.

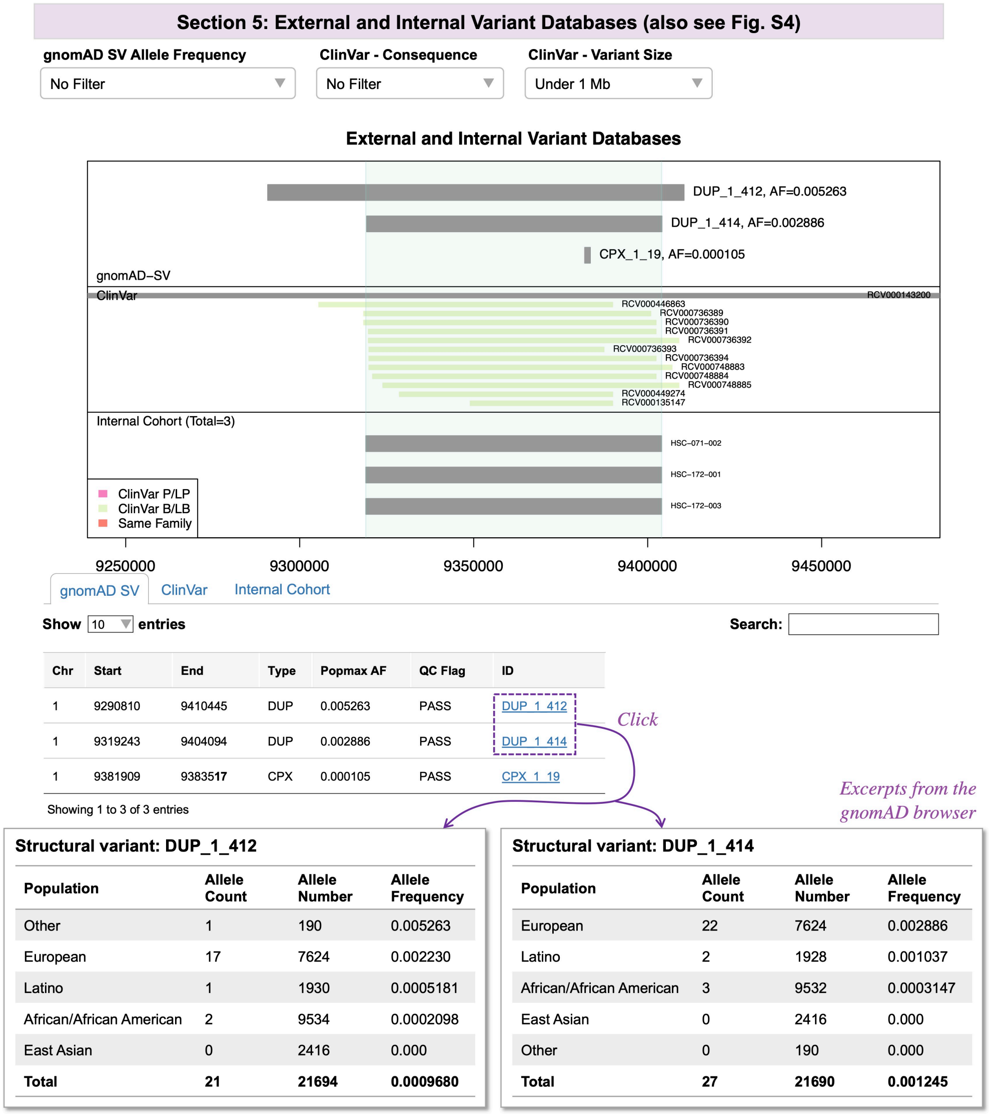

Figure S9. Use Case Scenario 3: Population Variation

The Internal and External Variant Databases section of the SCIP Visualization Module reveals that this duplication is similar in size to gnomAD-SV variant DUP_1_414 and is fully contained within another gnomAD-SV variant DUP_1_412. Collectively, these two variants were found in 48 samples. In addition, more than ten similar-sized B/LB ClinVar variants were found, without any P/LP variants.

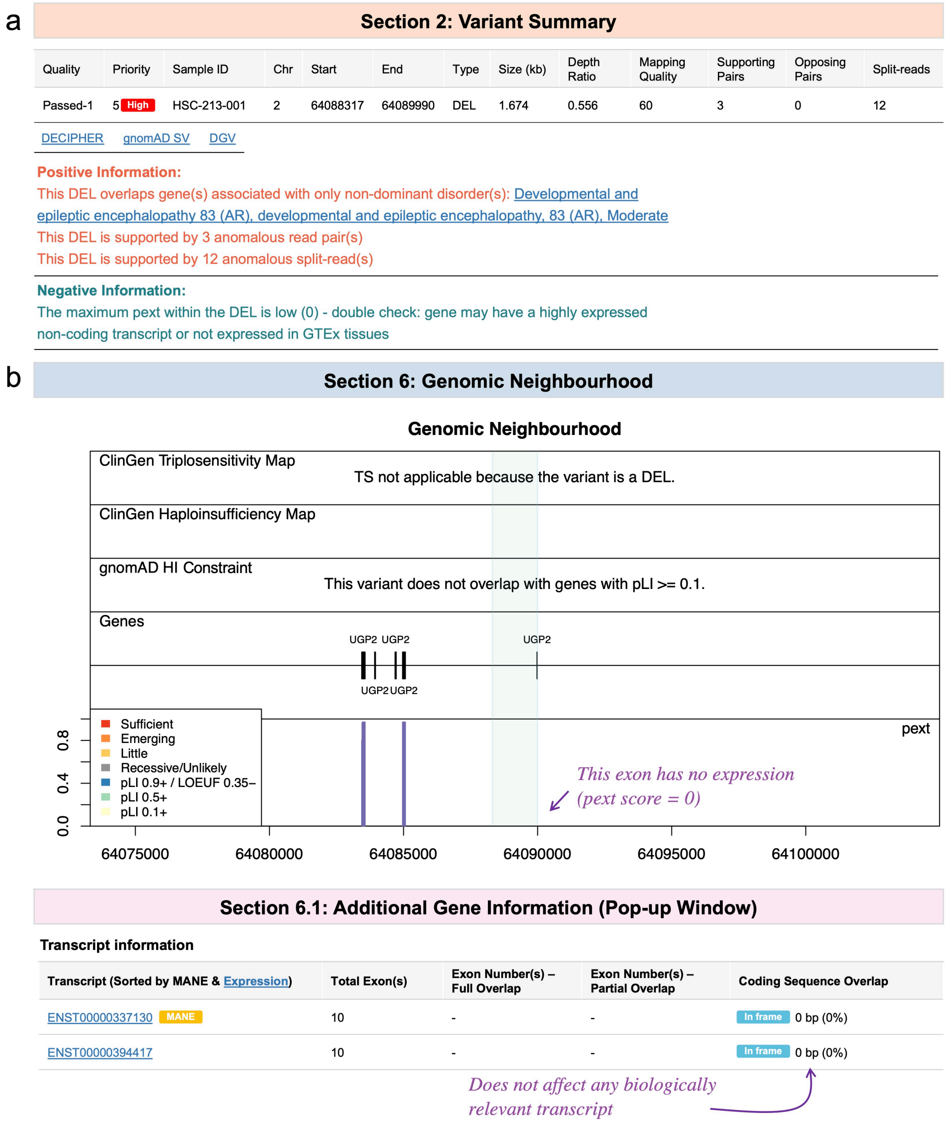

Figure S10. Use Case Scenario 4: Not Affecting a Biologically Relevant Transcript

This figure shows selected sections of the SCIP Visualization Module for a CNV excluded as not affecting a biologically relevant transcript. (a) The Variant Summary section indicates that the maximum pext (relative exon expression) score within this deletion is zero, i.e., no exons within this CNV were found to be expressed in any GTEx tissue. (b) The Genomic Neighbourhood section further confirms that the only exon overlapping this deletion has a zero pext score and is not on the MANE Select or any biologically relevant transcript. Comments are shown in purple.

Supplementary Tables

Table S1. P/LP and VUS CNVs for Evaluation of SCIP

A variant may appear multiple times if it was detected in multiple samples.

Chr	Start	End	Type	Inheritance	Interpretation
1	146460401	148468000	DEL	Maternal	P/LP
1	155460428	155495782	DEL	Unknown	P/LP
1	244763201	246458600	DEL	de novo	P/LP
2	10001	2082025	DEL	de novo	P/LP
2	1970583	1981286	DEL	Unknown	P/LP
2	50340806	50830652	DEL	de novo	P/LP
2	50383801	50503000	DEL	de novo	P/LP
2	50520342	50657518	DEL	de novo	P/LP
2	50527087	50773241	DEL	Paternal	P/LP
2	50574073	50661111	DEL	Unknown	P/LP
2	50615034	50855466	DEL	Maternal	P/LP
2	50615034	50855466	DEL	Maternal	P/LP
2	50895761	51038330	DEL	Unknown	P/LP
2	50899856	51181466	DEL	Unknown	P/LP
2	50908611	51143974	DEL	Unknown	P/LP
2	50923820	51049409	DEL	Unknown	P/LP
2	50949998	51062977	DEL	de novo	P/LP
2	50951514	51147537	DEL	Paternal	P/LP
2	50993730	51075017	DEL	Paternal	P/LP
2	147949949	148197893	DEL	de novo	P/LP
2	147987001	148166000	DEL	de novo	P/LP
2	152535795	158675354	DEL	de novo	P/LP
2	165284718	165483357	DEL	de novo	P/LP
2	179874281	184489810	DEL	de novo	P/LP
2	237530601	242141800	DEL	Unknown	P/LP
2	240641401	242152400	DEL	de novo	P/LP
4	112983777	113302123	DEL	de novo	P/LP
4	113366628	113370561	DEL	Paternal	P/LP
4	139347829	139351061	DEL	Unknown	P/LP
4	139347847	139351061	DEL	Unknown	P/LP
5	11801	4278200	DEL	Unknown	P/LP
5	11801	13809000	DEL	Unknown	P/LP
5	9226001	12664600	DEL	de novo	P/LP
5	10963840	11013399	DEL	Unknown	P/LP
5	11324695	11379314	DEL	Paternal	P/LP
5	88808127	89078617	DEL	de novo	P/LP
5	145818261	149866981	DEL	de novo	P/LP
6	33323870	33438436	DEL	de novo	P/LP
7	69982401	70173600	DEL	Maternal	P/LP
7	69982783	70173496	DEL	Maternal	P/LP
7	69982783	70173496	DEL	Maternal	P/LP
7	70314329	70738264	DEL	de novo	P/LP
7	70441029	70771836	DEL	de novo	P/LP
7	73235201	74734200	DEL	de novo	P/LP

7	105038832	105043479	DEL	Unknown	P/LP
7	152414801	159335800	DEL	de novo	P/LP
8	60001	6971800	DEL	de novo	P/LP
8	60001	8220594	DEL	de novo	P/LP
9	82919	11994940	DEL	de novo	P/LP
9	171401	9797000	DEL	de novo	P/LP
9	19567707	27573393	DEL	Unknown	P/LP
9	19684631	24685152	DEL	Unknown	P/LP
9	136029872	137168121	DEL	de novo	P/LP
9	139345895	139484281	DEL	Unknown	P/LP
10	6296201	11031800	DEL	Unknown	P/LP
10	74985960	74994405	DEL	Maternal	P/LP
11	70606001	70744400	DEL	Maternal	P/LP
11	126212847	135076600	DEL	de novo	P/LP
12	120438463	122740748	DEL	de novo	P/LP
13	35070057	35086088	DEL	de novo	P/LP
13	35622869	35660235	DEL	Unknown	P/LP
15	22609401	28415400	DEL	Unknown	P/LP
15	28682001	32393200	DEL	de novo	P/LP
15	28703201	32443600	DEL	Maternal	P/LP
15	30528001	32165400	DEL	de novo	P/LP
15	30558001	32464000	DEL	Unknown	P/LP
15	30582201	32163600	DEL	Unknown	P/LP
15	30588401	32153200	DEL	Unknown	P/LP
15	30592401	32153200	DEL	Unknown	P/LP
15	30625601	32163600	DEL	de novo	P/LP
15	57226482	57232892	DEL	Unknown	P/LP
15	82517801	84270000	DEL	Unknown	P/LP
15	92854397	92940464	DEL	de novo	P/LP
16	14688401	16394600	DEL	Maternal	P/LP
16	14798201	16304000	DEL	Maternal	P/LP
16	15030401	16307600	DEL	de novo	P/LP
16	15030401	16429200	DEL	Paternal	P/LP
16	15341801	18436400	DEL	Maternal	P/LP
16	28667201	29043400	DEL	Maternal	P/LP
16	28736001	29051600	DEL	de novo	P/LP
16	28805001	29040400	DEL	Maternal	P/LP
16	29538601	30189000	DEL	Unknown	P/LP
16	29540001	30188600	DEL	Unknown	P/LP
16	29540001	30188600	DEL	Unknown	P/LP
16	29540001	30188600	DEL	de novo	P/LP
16	29540001	30188600	DEL	de novo	P/LP
16	29544601	30188600	DEL	de novo	P/LP
16	29545601	30188600	DEL	de novo	P/LP
16	29552801	30188600	DEL	Unknown	P/LP
16	29555001	30188600	DEL	de novo	P/LP
16	29557801	30188600	DEL	Unknown	P/LP
16	49326510	53476612	DEL	de novo	P/LP
16	70203801	72582400	DEL	de novo	P/LP
16	89205401	89458400	DEL	de novo	P/LP

17	16845401	20516200	DEL	de novo	P/LP
17	29498538	29503050	DEL	de novo	P/LP
17	36228001	38250000	DEL	Unknown	P/LP
17	45625601	46135400	DEL	de novo	P/LP
18	112601	15375000	DEL	de novo	P/LP
18	66885569	80262919	DEL	Unknown	P/LP
19	11040058	11043883	DEL	Paternal	P/LP
19	13281027	13288239	DEL	Unknown	P/LP
20	67103	1358838	DEL	Maternal	P/LP
22	18897001	21388200	DEL	Unknown	P/LP
22	41113623	41129006	DEL	Unknown	P/LP
22	41990601	42490800	DEL	Unknown	P/LP
22	49654601	50660200	DEL	de novo	P/LP
22	50014902	50818468	DEL	de novo	P/LP
22	50246801	50800400	DEL	de novo	P/LP
22	50259048	50796600	DEL	de novo	P/LP
22	50683601	50764200	DEL	de novo	P/LP
X	11761754	11764267	DEL	de novo	P/LP
X	22646201	23812280	DEL	de novo	P/LP
X	27170601	31765200	DEL	de novo	P/LP
X	31926462	32095094	DEL	Unknown	P/LP
X	53395321	53416218	DEL	de novo	P/LP
X	53812201	54333200	DEL	Maternal	P/LP
X	53812201	54333200	DEL	Maternal	P/LP
X	63623511	63647786	DEL	Unknown	P/LP
X	78969585	155984200	DEL	Unknown	P/LP
X	155249293	155261376	DEL	Maternal	P/LP
1	143905001	149813000	DUP	Unknown	P/LP
1	146057001	149363000	DUP	Unknown	P/LP
1	146070001	148578000	DUP	Paternal	P/LP
1	146308001	148695000	DUP	Unknown	P/LP
1	146308001	148721000	DUP	Unknown	P/LP
1	146314001	148601000	DUP	Unknown	P/LP
1	146316001	148578000	DUP	Unknown	P/LP
1	146316001	149592000	DUP	Maternal	P/LP
1	146316001	149592000	DUP	Maternal	P/LP
1	146317001	148646000	DUP	Unknown	P/LP
1	146318001	148460000	DUP	Unknown	P/LP
1	146318001	148649000	DUP	de novo	P/LP
1	146367001	148718000	DUP	Unknown	P/LP
1	146985001	148599000	DUP	de novo	P/LP
2	95728001	113369000	DUP	de novo	P/LP
4	10001	9411000	DUP	de novo	P/LP
5	4283001	28797000	DUP	Unknown	P/LP
7	73113001	74414000	DUP	de novo	P/LP
7	73187001	74729000	DUP	de novo	P/LP
7	73192001	74754000	DUP	de novo	P/LP
7	73266001	74780000	DUP	Paternal	P/LP
9	135852001	137908000	DUP	Unknown	P/LP
10	65421001	72384000	DUP	de novo	P/LP

12	115255001	133257000	DUP	de novo	P/LP
14	102484001	106883800	DUP	de novo	P/LP
15	17499001	32128000	DUP	de novo	P/LP
15	18340001	28668000	DUP	de novo	P/LP
15	19563001	32217000	DUP	de novo	P/LP
15	19993001	28652000	DUP	de novo	P/LP
15	19996001	28667000	DUP	Maternal	P/LP
15	19996001	28668000	DUP	Maternal	P/LP
15	20100001	28789000	DUP	Unknown	P/LP
15	20187001	28601000	DUP	de novo	P/LP
15	20187001	28680000	DUP	Paternal	P/LP
15	22221001	32373000	DUP	de novo	P/LP
15	22221001	32473000	DUP	de novo	P/LP
15	22359001	28760000	DUP	de novo	P/LP
15	23180001	28391000	DUP	Maternal	P/LP
15	23428001	28667000	DUP	de novo	P/LP
15	23428001	32606000	DUP	de novo	P/LP
16	29432001	30285000	DUP	Unknown	P/LP
16	29437001	30291000	DUP	Unknown	P/LP
16	29437001	30296000	DUP	Maternal	P/LP
16	29549001	30285000	DUP	Unknown	P/LP
17	26936001	35060000	DUP	Unknown	P/LP
17	36321001	37983000	DUP	Unknown	P/LP
17	36369001	37921000	DUP	Paternal	P/LP
17	36459001	37902000	DUP	Unknown	P/LP
17	36459001	37921000	DUP	Maternal	P/LP
17	36459001	37921000	DUP	de novo	P/LP
17	41726001	43230000	DUP	de novo	P/LP
18	48731001	80263400	DUP	Unknown	P/LP
22	18933001	20340000	DUP	Paternal	P/LP
22	18933001	20597000	DUP	de novo	P/LP
22	18936001	21171000	DUP	Both	P/LP
22	18936001	21187000	DUP	de novo	P/LP
22	18938001	21182000	DUP	Maternal	P/LP
22	18939001	21080000	DUP	Paternal	P/LP
22	18939001	21114000	DUP	Paternal	P/LP
22	18939001	21169000	DUP	de novo	P/LP
22	18939001	21182000	DUP	Unknown	P/LP
22	18939001	21182000	DUP	Unknown	P/LP
22	18939001	21199000	DUP	Unknown	P/LP
22	18940001	21162000	DUP	Paternal	P/LP
X	154881001	155376000	DUP	Unknown	P/LP
X	154881001	155376000	DUP	Maternal	P/LP

Table S2. Exception List of Genes for which All Exons (Instead of Coding Exons Only) were Used by SCIP

Gene Symbol	Reason	Note
GJB1	Pathogenic noncoding variation (GeneReviews)	hg19 and hg38
GJC2	Pathogenic noncoding variation (GeneReviews)	hg19 and hg38
FOXP3	Pathogenic noncoding variation (GeneReviews)	hg19 and hg38
MBD5	Pathogenic noncoding variation (GeneReviews)	hg19 and hg38
TP53	Pathogenic noncoding variation (GeneReviews)	hg19 and hg38
PAX6	Pathogenic noncoding variation (GeneReviews)	hg19 and hg38
CFI	Pathogenic noncoding variation (GeneReviews)	hg19 and hg38
AMER1	Pathogenic noncoding variation (GeneReviews)	hg19 and hg38
FERMT1	Pathogenic noncoding variation (GeneReviews)	hg19 and hg38
MAPT-AS1	Pathogenic noncoding gene (SFARI)	hg19 and hg38
PTCHD1-AS	Pathogenic noncoding gene (SFARI)	hg38 only
RP11-40F8.2	Pathogenic noncoding gene (SFARI)	hg19 only
ABO	OMIM morbid, not marked as coding in Ensembl	hg19 only
GNAS-AS1	OMIM morbid, not marked as coding in Ensembl	hg19 and hg38
IGHG2	OMIM morbid, not marked as coding in Ensembl	hg19 and hg38
IGHM	OMIM morbid, not marked as coding in Ensembl	hg19 and hg38
IGKC	OMIM morbid, not marked as coding in Ensembl	hg19 and hg38
KCNQ1OT1	OMIM morbid, not marked as coding in Ensembl	hg19 and hg38
MIAT	OMIM morbid, not marked as coding in Ensembl	hg19 and hg38
MIR140	OMIM morbid, not marked as coding in Ensembl	hg19 and hg38
MIR184	OMIM morbid, not marked as coding in Ensembl	hg19 and hg38
MIR204	OMIM morbid, not marked as coding in Ensembl	hg19 and hg38
MIR2861	OMIM morbid, not marked as coding in Ensembl	hg38 only
MIR96	OMIM morbid, not marked as coding in Ensembl	hg19 and hg38
NUTM2B-AS1	OMIM morbid, not marked as coding in Ensembl	hg38 only
PCBP1-AS1	OMIM morbid, not marked as coding in Ensembl	hg19 and hg38
PLK1S1	OMIM morbid, not marked as coding in Ensembl	hg19 only
RMRP	OMIM morbid, not marked as coding in Ensembl	hg19 and hg38
RNU4ATAC	OMIM morbid, not marked as coding in Ensembl	hg19 and hg38
RNU7-1	OMIM morbid, not marked as coding in Ensembl	hg19 and hg38
RP11-773D16.1	OMIM morbid, not marked as coding in Ensembl	hg19 only
SNORA31	OMIM morbid, not marked as coding in Ensembl	hg19 and hg38
SNORD118	OMIM morbid, not marked as coding in Ensembl	hg19 and hg38
SYN2	OMIM morbid, not marked as coding in Ensembl	hg19 only
TRAC	OMIM morbid, not marked as coding in Ensembl	hg19 and hg38
TTC25	OMIM morbid, not marked as coding in Ensembl	hg19 only
Telomerase-vert	OMIM morbid, not marked as coding in Ensembl	hg38 only
TERC	OMIM morbid, not marked as coding in Ensembl	hg19 only
XIST	OMIM morbid, not marked as coding in Ensembl	hg19 and hg38

Table S3. Files Required by the SCIP Prioritization Module and Corresponding Handles in the Prioritization Module Configuration File

The SCIP Prioritization Module configuration file is in plain text, named pipeline_config.txt or pipeline_config_hg38.txt, and placed in the system running the SCIP backend. Note that this is different from the Visualization Module configuration file (Table S5).

File Handle	Description	Notes
From external resources and databases		
gnomAD_SV	gnomAD-SV sites VCF file	The corresponding TBI index file is also required.<br>hg19 version (gnomad_v2.1_sv.sites.vcf.gz) and its TBI index are available on gnomAD website.<br>(https://gnomad.broadinstitute.org/downloads)<br>hg38 version was generated in-house and is provided.
gnomAD_constraints	gnomAD pLOF metrics by transcript TSV file	gnomad.v2.1.1.lof_metrics.by_transcript.txt.bgz, available on gnomAD website (link above).<br>Same file for hg19 and hg38.
gnomAD_pext	Maximum pext score across tissues by position	The corresponding TBI index file is also required.<br>Generated in-house and is provided.<br>Different files for hg19 and hg38.
gnomAD_common	gnomAD-SV deletions and duplications with popmax at least 1%, genes fully contained within them	Generated in-house and is provided.<br>Different files for hg19 and hg38.
ClinGen_dosage_region	ClinGen dosage sensitivity map: region curation list TSV file	ClinGen_region_curation_list_[GRCh37/38].tsv<br>ClinGen_gene_curation_list_[GRCh37/38].tsv<br>**Important: we recommend updating this file at least quarterly. The latest files are available at the ClinGen FTP: https://ftp.clinicalgenome.org/. Update the configuration file to specify the name of the new file.**
ClinGen_dosage_gene	ClinGen dosage sensitivity map: gene curation list TSV file	
ClinVar_CNV	Processed ClinVar CNV information	Generated in-house and is provided.<br>This file may be periodically updated.<br>Different files for hg19 and hg38.<br>**Important: we recommend updating this file at least quarterly. See the SCIP GitHub site for details. Update the configuration file to specify the name of the new file.**
OMIM	OMIM genemap2 TXT file	This file may be directly obtained from OMIM and is periodically updated.<br>Request access at https://omim.org/downloads<br>**Important: we recommend updating this file at least quarterly.**
GenCC	GenCC gene curations	Generated in-house and is provided.<br>GenCC may periodically update the data.<br>Same file for hg19 and hg38.<br>**Important: we recommend updating this file at least quarterly. See the SCIP GitHub site for details. Update the configuration file to specify the name of the new file.**
coding_exons	Coding exon coordinates, by gene	Generated in-house and are provided.<br>Different files for hg19 and hg38.
coding_exons_plotting	Coding exon coordinates, by gene, optimized for plotting	

`all_exons`	Intron-exon junction coordinates	
`transcript_info`	Transcript information by gene, for biologically relevant transcripts only	
`gene_strand`	Strand information of genes	Obtained from Ensembl BioMart and is provided.<br>Different files for hg19 and hg38.
`REF_BAM`	Whole-genome alignment file for a reference sample (e.g., NA12878)	hg19: file `NA12878_S1.bam` available at ftp://ftp.sra.ebi.ac.uk/vol1/run/ERR194/ERR194147<br>hg38: file `NA12878.final.cram` available at ftp://ftp.sra.ebi.ac.uk/vol1/run/ERR323/ERR3239334<br>The files must be accessible to `samtools`. Thus, it is required to generate BAI/CRAI index files. Based on our experience, it may be necessary to convert the hg38 cram to bam file for SCIP usage.
`noncoding`	Exception list, see Table S2	Generated in-house and is provided.<br>Same file for hg19 and hg38.<br>**Important: we recommend updating this file if new pathogenic non-coding regions are discovered. See the SCIP GitHub site for details. Update the configuration file to specify the name of the new file.**
`lowqual_reg`	Low-quality regions	Generated in-house and is provided.<br>Recurrent breakpoints file available for hg19 only. For hg38, please specify path to an empty file.<br>Different files for hg19 and hg38.<br>See Supplementary Methods (Recurrence Regions and Recurrent Breakpoints section) for details.
`lowqual_bkp`	Recurrent breakpoints	
Files to be provided by the user		
`SAMPLE_ID`	Sample IDs and corresponding names of alignment files	Two tab-separated columns.<br>Each row is a sample. The second column is sample ID; the first column is the name prefix of the alignment file for this sample.<br>For example, if alignment file for sample SAM-001-001 is alignment001.bam, specify the following line in this file:<br>`alignment001` | `SAM-001-001`
`cohort_CNV`	CNVs detected in the internal cohorts	Six tab-separated columns.<br>The first four columns are chromosome, start, end, CNV type (DEL or DUP); the fifth column is sample ID, and the sixth column is algorithm used to identify this variant. For example:<br>`1` | `10001` | `50001` | `DUP` | `SAM-001-001` | `ERDS`<br>To allow identification of members of the same family, sample IDs must follow this format: `[three-letter prefix]-[3 or 4 digits family ID]-[family member ID]`. For family member ID, we recommend using 001 for proband, 002 for mother, 003 for father, and use 004+ for other family members.<br>Optional. If a user does not wish to provide this file, please specify the path to an empty file instead.
`gene_interest`	List of candidate genes	One column, HGNC gene symbols.<br>Specify candidate genes based on your disease(s)-of-interest. CNVs overlapping these genes will be given a clinical relevance score of 2 (Figure S3).<br>Optional. If a user does not wish to provide this file, please specify the path to an empty file instead.

<table>
<tr><td><code>search_terms</code></td><td>Search terms, for the Genes table in Section 6 of the SCIP Visualization Module</td><td>Two tab-separated columns.
The first column is the search term (for performing the Google search), based on your disease(s)-of-interest, and the second column is its abbreviation (for display). For example:
<table><tr><td><code>developmental delay</code></td><td><code>DD</code></td></tr></table>If abbreviation is not needed, the two columns can be identical.
Optional. If a user does not wish to provide this file, please specify the path to an empty file instead.</td></tr>
<tr><td><code>GO_terms</code></td><td>Relevant GO terms for each gene</td><td>Two tab-separated columns.
Depending on the disease(s)-of-interest, the user may identify relevant GO terms. In this file, each row is a gene that contains at least one of the relevant GO terms. The first column is HGNC gene symbol, and the second column is a list of GO terms for this gene, separated by a vertical bar (|). For example:
<table><tr><td><code>DGKA</code></td><td><code>Signaling|CalciumIon</code></td></tr></table>Optional. If a user does not wish to provide this file, please specify the path to an empty file instead.</td></tr>
<tr><td><code>expression_file</code></td><td>Expression level per gene in the tissue-of-interest</td><td>Two tab-separated columns.
The first column is HGNC gene symbol, and the second column is expression level. For example:
<table><tr><td><code>A1BG</code></td><td><code>7.627</code></td></tr></table>Optional. If a user does not wish to provide this file, please specify the path to an empty file instead.</td></tr>
<tr><td colspan="3">User-specified parameters</td></tr>
<tr><td><code>ALIGNMENT_PATH</code></td><td colspan="2">Absolute path to the directory containing alignment files. A sub-directory should exist for each sample, with the name specified in the <code>SAMPLE_ID</code> file. For example, if the following line was included in the SAMPLE_ID file:
<table><tr><td><code>alignment001</code></td><td><code>SAM-001-001</code></td></tr></table>The alignment file for SAM-001-001 should be found at:
<code>[ALIGNMENT_PATH]/alignment001/alignment001.bam</code> (or <code>cram</code>)</td></tr>
<tr><td><code>PREFIX</code></td><td colspan="2">Not used in typical situations, please specify as NA.</td></tr>
<tr><td><code>CURRENT_PATH</code></td><td colspan="2">Absolute path to the current directory, which contains this configuration file and all scripts for the SCIP Prioritization Module.</td></tr>
</table>

Table S4. Full List of Possible Positive and Negative Information Displayed in Part 2 of the SCIP Visualization Interface

**Positive Information**
This DEL is fully contained in ClinGen HI region(s)
This DEL overlaps ClinGen HI gene(s)
This DEL/DUP overlaps candidate gene(s)
This DEL overlaps gene(s) with pLI >= 0.9 and/or LOEUF <= 0.35
This DEL overlaps gene(s) with pLI >= 0.5 but < 0.9
This DEL overlaps gene(s) associated with dominant disorder(s)
This DEL overlaps gene(s) associated with only non-dominant disorder(s)
This DUP fully contains ClinGen TS region(s) or gene(s)
This DUP overlaps ClinGen TS gene(s)
This intragenic DUP overlaps ClinGen HI gene(s)
This intragenic DUP overlaps gene(s) with pLI >= 0.9 and/or LOEUF <= 0.35
This intragenic DUP overlaps gene(s) with pLI >= 0.5 but < 0.9
This intragenic DUP overlaps gene(s) associated with dominant disorder(s)
This intragenic DUP overlaps gene(s) associated with only non-dominant disorder(s)
This non-intragenic DUP overlaps gene(s) associated with dominant disorder(s)
This non-intragenic DUP overlaps gene(s) associated with only non-dominant disorder(s)
This DEL/DUP is supported by [number] anomalous read pair(s)
This DEL/DUP is supported by [number] anomalous split-read(s)
**Negative Information**
This DEL/DUP has [number]% overlap with gnomAD DEL/DUP(s) with popmax >= 1%
This DEL/DUP has both breakpoints within 10 bp of an intron-exon junction – rule out pseudogene insertion
This DEL/DUP has [number]% overlap with ERDS+ DEL/DUP recurrence region(s) or other low quality region(s)
This DEL/DUP has both breakpoints within 5000 bp of Manta DEL/DUP recurrence region(s)
The maximum pext within the DEL/DUP is low ([number]) – double check: gene may have a highly expressed non-coding transcript or not expressed in GTEx tissues
There are no anomalous read(s) supporting this DEL/DUP

Table S5. Example Configuration File for the SCIP Visualization Module

This file is in plain text format, named interface_config.txt, and placed in the system running the SCIP Visualization Module.

`LIST_NAME`	Name of the sample to be analyzed, e.g., MSG-0001-001
`TEMP_FILE_DIR`	Directory containing the files generated by the SCIP backend
`ROOT_DIR`	Directory containing this file and the SCIP Visualization Module R script
`USER`	Optional, name of the user, used to track interpretations across users

Table S6. Cases Analyzed by the Head-to-head Comparison

Case ID	Chr	Start	End	Type	Size (kb)	Reviewable CNVs (SCIP)	P/LP Rank (SCIP)
MSG-2009-001	19	11040058	11043883	DEL	3.83	4	1
MSG-3698-004	17	29498538	29503050	DEL	4.51	5	2
MSG-2279-001	13	35070057	35086088	DEL	16.03	5	1
MSG-4097-001	10	74985960	74994405	DEL	8.45	1	1
MSG-3256-001	7	69982783	70173496	DEL	190.71	14	1
MSG-2959-001	15	92854397	92940464	DEL	86.07	1	1
MSG-2983-001	22	50014902	50806000	DEL	803.57	2	1
MSG-4058-001	16	29555001	30188600	DEL	633.60	2	1
MSG-1823-001	20	67103	1358838	DEL	1,291.74	5	1
MSG-3643-004	15	30528001	32165400	DEL	1,637.40	6	1
MSG-2717-001	16	29437001	30296000	DUP	859.00	2	1
MSG-3993-001	17	36369001	37921000	DUP	1552.00	2	2
MSG-4065-001	7	73003001	74754000	DUP	1751.00	1	1
MSG-3418-004	No finding					2	N/A
MSG-4047-004	No finding					1	N/A

Supplementary References

GTEx Consortium (2017) Genetic effects on gene expression across human tissues. Nature 550: 204-213. doi: 10.1038/nature24277

Howe KL, Achuthan P, Allen J, Allen J, Alvarez-Jarreta J, Amode MR, Armean IM, Azov AG, Bennett R, Bhai J, Billis K, Boddu S, Charkhchi M, Cummins C, Da Rin Fioretto L, Davidson C, Dodiya K, El Houdaigui B, Fatima R, Gall A, Garcia Giron C, Grego T, Guijarro-Clarke C, Haggerty L, Hemrom A, Hourlier T, Izuogu OG, Juettemann T, Kaikala V, Kay M, Lavidas I, Le T, Lemos D, Gonzalez Martinez J, Marugán JC, Maurel T, McMahon AC, Mohanan S, Moore B, Muffato M, Oheh DN, Paraschas D, Parker A, Parton A, Prosovetskaia I, Sakthivel MP, Salam AIA, Schmitt BM, Schuilenburg H, Sheppard D, Steed E, Szpak M, Szuba M, Taylor K, Thormann A, Threadgold G, Walts B, Winterbottom A, Chakiachvili M, Chaubal A, De Silva N, Flint B, Frankish A, Hunt SE, Iisley GR, Langridge N, Loveland JE, Martin FJ, Mudge JM, Morales J, Perry E, Ruffier M, Tate J, Thybert D, Trevanion SJ, Cunningham F, Yates AD, Zerbino DR, Flicek P (2021) Ensembl 2021. Nucleic acids research 49: D884-D891. doi: 10.1093/nar/gkaa942

Quinlan AR, Hall IM (2010) BEDTools: a flexible suite of utilities for comparing genomic features. Bioinformatics (Oxford, England) 26: 841-842. doi: 10.1093/bioinformatics/btq033

The International Batten Disease Consortium (1995) Isolation of a novel gene underlying Batten disease, CLN3. The International Batten Disease Consortium. Cell 82: 949-57. doi: 10.1016/0092-8674(95)90274-0
